# A novel microbial and hepatic biotransformation-integrated network pharmacology strategy explores the therapeutic mechanisms of bioactive herbal products in neurological diseases: the effects of Astragaloside IV on intracerebral hemorrhage as an example

**DOI:** 10.1186/s13020-023-00745-5

**Published:** 2023-04-17

**Authors:** En Hu, Zhilin Li, Teng Li, Xueping Yang, Ruoqi Ding, Haoying Jiang, Hong Su, Menghan Cheng, Zhe Yu, Haigang Li, Tao Tang, Yang Wang

**Affiliations:** 1 Department of Integrated Traditional Chinese and Western Medicine, Institute of Integrative Medicine, Xiangya Hospital, Central South University, Changsha, Hunan People’s Republic of China 410008; 2 National Clinical Research Center for Geriatric Disorders, Xiangya Hospital, Central South University, Changsha, Hunan People’s Republic of China 410008; 3 Hunan Key Laboratory of the Research and Development of Novel Pharmaceutical Preparations, Changsha Medical University, Changsha, Hunan People’s Republic of China 410219

**Keywords:** Herbal products, Gut microbiota, Biotransformation, Liver, Network pharmacology, Astragaloside IV, Intracerebral hemorrhage, Cycloastragenol, 3-*epi*-cycloastragenol, Microglia

## Abstract

**Background:**

The oral bioavailability and blood–brain barrier permeability of many herbal products are too low to explain the significant efficacy fully. Gut microbiota and liver can metabolize herbal ingredients to more absorbable forms. The current study aims to evaluate the ability of a novel biotransformation-integrated network pharmacology strategy to discover the therapeutic mechanisms of low-bioavailability herbal products in neurological diseases.

**Methods:**

A study on the mechanisms of Astragaloside IV (ASIV) in treating intracerebral hemorrhage (ICH) was selected as an example. Firstly, the absorbed ASIV metabolites were collected by a literature search. Next, the ADMET properties and the ICH-associated targets of ASIV and its metabolites were compared. Finally, the biotransformation-increased targets and biological processes were screened out and verified by molecular docking, molecular dynamics simulation, and cell and animal experiments.

**Results:**

The metabolites (3-*epi*-cycloastragenol and cycloastragenol) showed higher bioavailability and blood–brain barrier permeability than ASIV. Biotransformation added the targets ASIV in ICH, including PTK2, CDC42, CSF1R, and TNF. The increased targets were primarily enriched in microglia and involved in cell migration, proliferation, and inflammation. The computer simulations revealed that 3-*epi*-cycloastragenol bound CSF1R and cycloastragenol bound PTK2 and CDC42 stably. The In vivo and in vitro studies confirmed that the ASIV-derived metabolites suppressed CDC42 and CSF1R expression and inhibited microglia migration, proliferation, and TNF-α secretion.

**Conclusion:**

ASIV inhibits post-ICH microglia/macrophage proliferation and migration, probably through its transformed products to bind CDC42, PTK2, and CSF1R. The integrated strategy can be used to discover novel mechanisms of herbal products or traditional Chinses medicine in treating diseases.

**Graphical Abstract:**

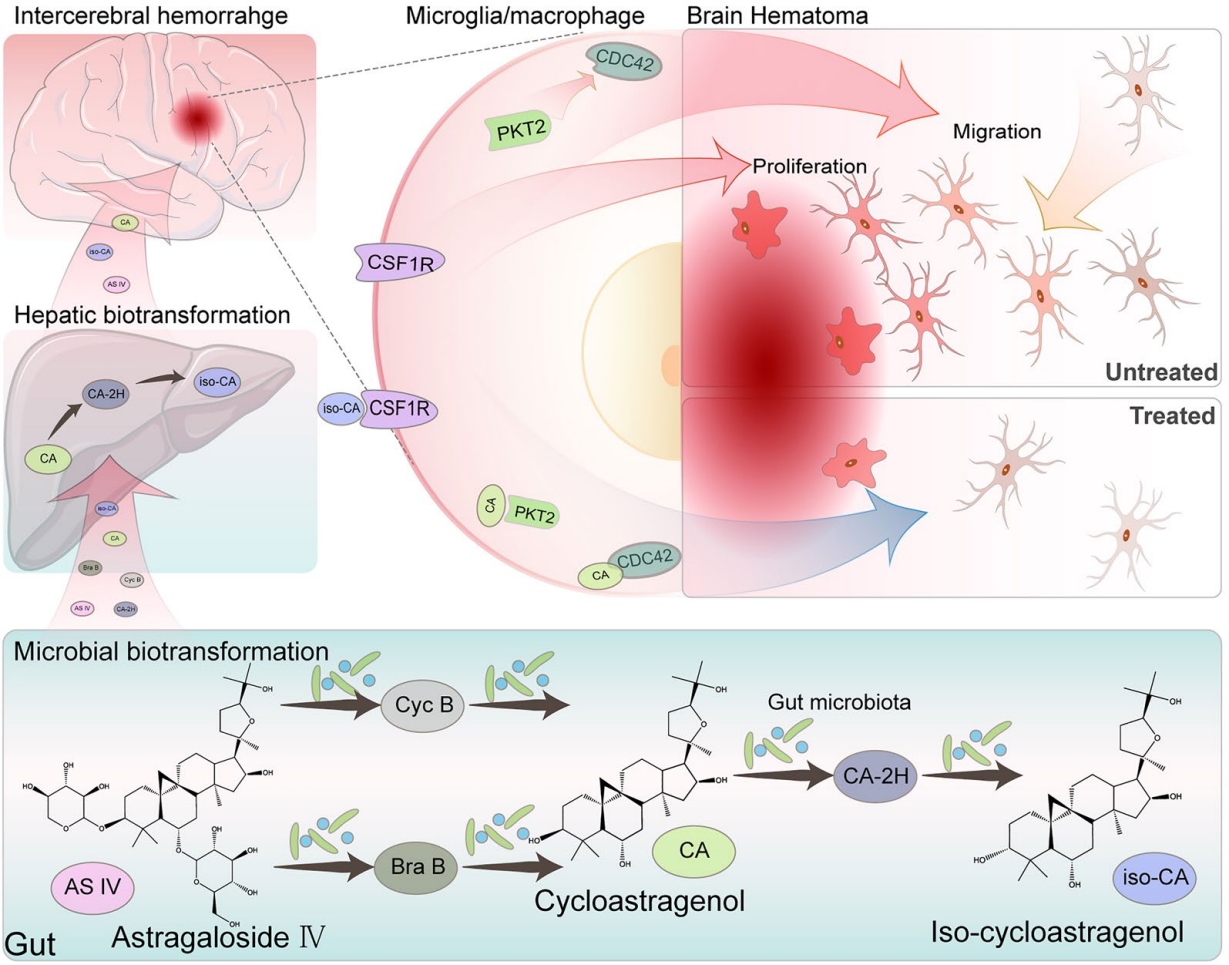

**Supplementary Information:**

The online version contains supplementary material available at 10.1186/s13020-023-00745-5.

## Introduction

Herbal products are compounds produced by herbs. These ingredients are the primary sources of drugs. Statistically, herbal products or their derivatives constitute ~ 34% of all the Food and Drug Administration (FDA) approved small-molecule drugs [1]. Besides the approved natural medicines, numerous herbal products exert therapeutic effects on diverse diseases through varying targets [2]. However, the therapeutic mechanisms and targets of most herbal products remain uncovered.

Network pharmacology is often used to explore the potential targets and therapeutic mechanisms of herbal products or formulae [3, 4]. This method screens the potential targets primarily based on the direct ligand-receptor interactions [4]. However, absorption, distribution, metabolism, excretion, and toxicity (ADMET) properties of many herbal products are too poor to bind the putative targets in the lesions sufficiently, especially in the brain [5–7]. In this regard, most studies exclude the ingredients with low oral bioavailability (OB), drug-likeness (DL), or blood–brain barrier (BBB) permeability to reduce the false positive results in network pharmacology [8–12]. However, this strategy ignored the therapeutic effects and mechanisms of the low-OB compounds. In addition, some studies manually re-enrolled the reported functional compounds as input [8–10]. This method considered the limited compound-target interactions, but it may overstate the actual actions of the scant complex. Therefore, it is critical to establish a new strategy to tailor the input of low-OB herbal products in network-pharmacology studies.

The rapid development of microbiology in recent years has highlighted the roles of gut microbiota in transforming unabsorbed herbal products [5–7]. When orally administrated, the low-OB herbal products may unavoidably come into contact with and be metabolized by the gut microbiota. Some resulting products can access the bloodstream and the targeted organs sequentially [6]. For instance, ginsenoside Rb1 works only after the gut microbiota metabolizes to hydrophobic ginsenoside CK [13]. Moreover, many compounds undergo hepatic metabolism before entering their targeted organs [14]. Thus, microbial- and hepatic-biotransformation analyses may add information to explain the actions of the poorly-absorbed herbal products.

Intracerebral hemorrhage (ICH) is a notorious subtype of stroke. Though representing only 15% of all strokes, ICH accounts for 50% of stroke-related deaths, leaving enormous social and economic burdens [15, 16]. Worse still, few therapeutic methods are currently proven effective in ICH patients [15]. Fortunately, several herbal products and traditional Chinese medicine (TCM) have shown considerable efficacy in preclinic studies, such as Astragaloside IV (ASIV) [17, 18]. However, the BBB hampers drugs from accessing brain parenchyma. The discrepancy between the significant efficacy and the low BBB permeability makes it necessary to consider other ways like microbial and hepatic biotransformation when exploring the therapeutic mechanisms of herbal medicines in ICH. ASIV is one of the main bioactive ingredients in the widely prescribed TCM, *Astragali Radix*, in ICH patients [17]. Previous studies have revealed the neuroprotective effect of ASIV in ICH and other neurological diseases [17, 19, 20]. However, the reported OB of ASIV is low, 2.2–3.7% in rats and 7.4% in dogs, and the gastrointestinal permeability is also weak [21, 22]. It suggests that ASIV may act in specific forms other than the original conformation. The oral-administrated ASIV can easily contact gut microbiota and then be transformed into secondary metabolites such as cycloastragenol (CA) and 3-*epi*-cycloastragenol (iso-CA) [21, 22]. The absorbed parts undergo liver metabolism. Therefore, some metabolites are detected in blood [21, 22] and exert neuroprotective effects in post-stroke mice [23, 24]. Thus, the microbial and hepatic biotransformation shall be taken into account for ASIV to treat ICH.

**Fig. 1:**
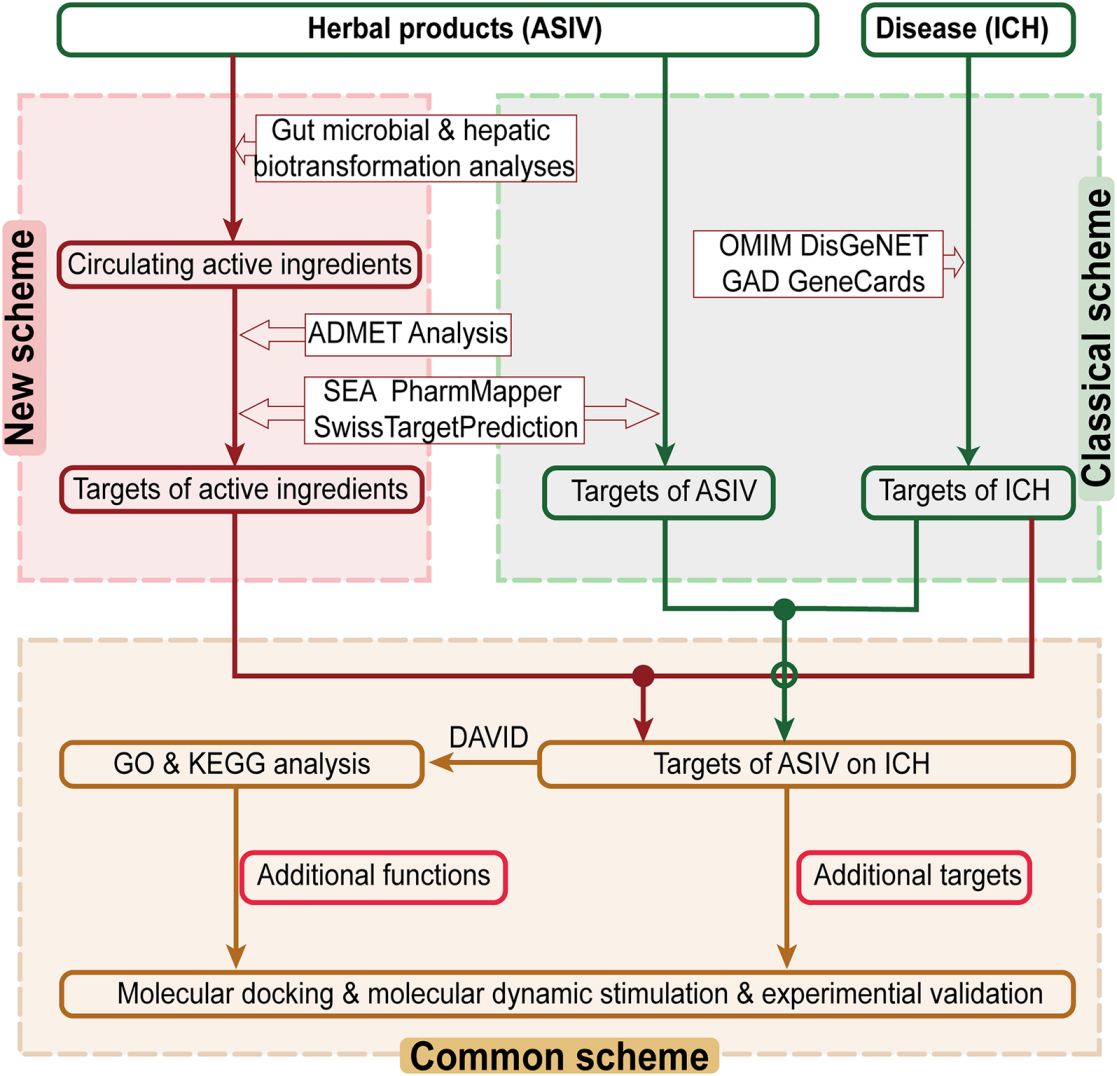
Comparison of the new strategy with the classical scheme. Gut microbial- and hepatic-biotransformation analyses are performed before the target prediction of herbal products. Next, the ASIV targets after biotransformation are intersected with that of ICH. Then, GO and KEGG analyses enrich the related biological processes. At last, molecular docking, molecular dynamics simulation, and cell and animal experiments confirm the transformation-added targets and pathways. *ASIV* Astragaloside IV, *ICH* intracerebral hemorrhage. Solid circle: intersected; hollow circle: not intersected

In this study, we choose ASIV as an example to explore the therapeutic mechanisms of low-OB herbal products in neurological diseases using a novel network pharmacology-based strategy. In this strategy, the gut microbiota- and liver-transformed metabolites of herbal products are inputted into the network pharmacology scheme. As a result, biotransformation-added targets and biological processes are found. Moreover, the biotransformation-enhanced effects are further verified by molecular docking, molecular dynamics simulation, and cell/animal experiments (Fig. 1).

## Material and methods

### Inclusion of the potentially effective compounds of ASIV

The detectable derivatives of ASIV in the bloodstream were enrolled according to the literature [21, 22, 25], including ASIV and the products after gut microbial or hepatic biotransformation.

### Prediction of ADMET properties

The ADMET properties of the candidate effective compounds were predicted by admetSAR2.0 (http://lmmd.ecust.edu.cn/admetsar2/) [26] and SwissADME (http://www.swissadme.ch/) [27]. SMILEs were loaded into these databases. Lipophilicity was predicted by the iLOGP method [28]. Gastrointestinal (GI) absorption was calculated according to the white/yolk of the BOILED-Egg [29]. BBB permeability was obtained from admetSAR2.0 [26]. In addition, DL was judged by the Lipinski rule [27].

### Targets screening of potentially effective compounds

The targets of potentially effective compounds were predicted by Similarity Ensemble Approach (SEA, http://sea.bkslab.org/, threshold: Max Tc > 0.57) [30], SwissTargetPrediction (http://www.swisstargetprediction.ch/, threshold: known actives (2D/3D) > 1) [31], and PharmMapper (http://www.lilab-ecust.cn/pharmmapper/, threshold: fit score > 2.0) [32]. Then, the resulting targets were uniformed to gene names by UniPort (https://www.uniprot.org/). The non-human genes were removed. Next, a compound-target network was constructed by the Cytoscape software (Version 3.9.2). This network displayed the shared and additional targets after microbial and hepatic transformation.

### Screening of ICH targets

The ICH-related genes were gathered by searching “intracerebral hemorrhage”, “brain hemorrhage”, “hemorrhagic stroke”, “cerebral hemorrhage”, and “stroke, hemorrhagic” in OMIM (https://omim.org/), DisGeNET (http://www.disgenet.org/, threshold: GDA score > 0.1) [33], Genetic Association Database (GAD, http://geneticassociationdb.nih.gov/) [34], and GeneCards (https://www.genecards.org/, threshold: relevance > 10) [35]. The maximum scores were selected for genes with multiple results in each database.

### Protein–protein interaction (PPI) network

The intersected targets of compounds and diseases were imputed to STRING (https://string-db.org/) with the default parameters. The resulting networks were uploaded to Cytoscape for further visualization. Three networks were constructed, including the ASIV targets-ICH, post-transformation targets-ICH, and increased targets-ICH networks. Then, the subnetwork was analyzed by the MCODE plugin in Cytoscape.

### Pathway analysis

To further explore the importance of microbial and hepatic transformation in treating diseases, GO enrichment and KEGG pathway analyses were performed using DAVID (https://david.ncifcrf.gov/). The biotransformation-added ICH targets were analyzed. The top 10 (ranked by *P* value) biological processes, cellular compounds, and molecular functions from GO and the top 30 pathways (ranked by P value), “Human Disease” excluded, from KEGG analyses were displayed. The results were visualized by the SangerBox website (http://sangerbox.com/).

### The cell-specific gene distribution analysis

The biotransformation-increased targets were inputted into the Brain RNA-Seq database (http://www.brainrnaseq.org/). Then, the Fragments Per Kilobase of exon model per Million mapped fragments (FPKM) of all inputs in *Homo sapiens* were added to obtain the primarily enriched cell types [36].

### Molecular docking

The SMILEs of the potential active compounds were gained from Chemdraw (Chemdraw Ultra 8.0, Cambridge Soft, USA). Then, Chem 3D Pro was employed to minimize the energy through the molecular mechanics-2 force field. Protein structures were gathered from RCSB Protein Data Bank (www.rcsb.org). Water and hetero molecules were removed, and hydrogen atoms were added by AutoDock tools (1.5.6). The active center of the protein was predicted by DeepSite (https://playmolecule.com/deepsite/). A 30 × 30 × 30 Ǻ (x, y, z) grid was set, with a grid spacing of 0.375 Ǻ. Then, autoDock vina was used to analyze the binding mode between ligands and receptors. The conformations with the lowest binding free energy were selected as the most potential binding models. At last, the docked structures were analyzed and visualized by PyMol 2.3.0 (http://www.pymol.org/) and Protein–Ligand Interaction Profiler (https://plip-tool.biotec.tu-dresden.de).

### Molecular dynamics simulation

GROMACS (version 2019.6) was used for molecular dynamics simulation. The AnteChamber Python Parser interface in AmberTools was adopted to parameterize the topologies, atomic types, and charges of small molecules. Amber99sb-ildn force field was applied for all simulations. SPC216 water molecules were added to the dodecahedral box. Then, Na^+^ and Cl^–^ were added to balance the charge to neutral. Afterward, energy was minimized. And the system was heated to 37.0℃ with a time step of 1 fs during the 200 ps-simulation in a constant Number of particles, Volume, and Temperature (NVT) method. Then, in the 100 ps-constant Number of particles, Pressure, and Temperature (NPT) simulation, the system was balanced to one atmospheric pressure with a time step of 2 fs. The pressure and temperature were adjusted by the V-rescale thermostat. The pressure was also adjusted by the Parrinello-Rahman. The molecular dynamics simulation was run for 80 ns with time steps of 2 fs. The 10 ps-interval trajectories were saved for analysis.

The binding free energy was evaluated by using the molecular Mechanics-Poisson-Boltzmann/surface area method. The 20 trajectories from the 50–55 ns of simulation at intervals of 250 ps were chosen to calculate the free energy using the g_mmpbsa method.

### Cell culture

BV2 (YBC061, ybio Biotechnology, Shanghai, China), a mouse-derived microglial cell line, was cultured with high-glucose Dulbecco’s modified Eagle’s medium (DMEM, C11995500BT, Gibco, Grand Island, NY, USA), containing 10% fetal bovine serum (SA211.02, CellMax, Beijing, China), 100 U/ml penicillin, and 100 g/ml Streptomycin in a 5% CO_2_ incubator (37 °C).

### Cell viability

Cell survival was assayed by cell counting kit-8 (CCK8) according to the manufacturer’s instructions (C0038, Beyotime Biotechnology, Beijing, China). For toxicity detection, five thousand cells were plated into 96-well plates. Then, the logarithmic-phase cells were treated with ASIV (0, 5, 10, 20, 40, 80, or 160 μM) or CA (0, 0.2, 1, 5, 10, 20, or 40 μM) for 6 h. For proliferation assay, two thousand cells were plated into a 96-well plate upon logarithmic growth phase. LPS (1 μg/ml), LPS + ASIV (10, 20, or 40 μM), or LPS + CA (1, 5, or 10 μM) were added for 6 h. Then, CCK-8 solution was supplemented to each well. A 1-h incubation at 37 °C was proceeded before measuring the absorbance at 450 nm with a Microplate photometer (Thermo, USA).

### Cell migration assay

Cell migration ability was detected with wound healing assay. BV2 cells were seeded in a six-well plate. When the cells fused into a monolayer, a 10-µl pipette tip perpendicular was used to scratch on the bottom of the well. The plate was washed twice to remove the detached cells. Then, the cells were treated with LPS (1 μg/ml), LPS + ASIV (10, 20, or 40 μM), or LPS + CA (1, 5, or 10 μM) for 24 h. Images were captured at 0 h and 24 h. The wound area was analyzed by Image J (National Institutes of Health). The migration rates were calculated as the percentage of the scratch closed after 24 h. The formula was: % scratch closed = (scratch area (0 h) − scratch area (24 h))/scratch area (0 h) × 100.

### Animal experiments

Male C57 BL/6 mice (12 weeks) were obtained from the Hunan Slake Jingda Laboratory Animal Co., Ltd. (Changsha, China) and housed according to the Animals (Scientific Procedures) Act 1986. All protocols were approved by the Medical Ethics Committee of Central South University (CSU-2022-0168).

Mice were cohoused for one week before surgery. They were randomly assigned into five groups: Sham, ICH, low dose (ASIV-L, 25 mg/kg), median dose (ASIV-M, 50 mg/kg), and high dose (ASIV-H, 100 mg/kg). ASIV was purchased from Source Leaf Biological Co., LTD (S31401, purity: 99.8%, Shanghai, China). ICH was induced by collagenase injection (type VII, 0.075 unit in 0.5 μl, C0773, sigma, St. Louis, MO, USA) into the right globus pallidum (coordinate: 0.5 mm posterior, 2.0 mm lateral to the bregma and 4.0 mm ventral to the skull surface). After being operated, the animals were administrated with ASIV (suspended in distilled water) or an equal volume of distilled water by gavage daily for three days.

On the 3rd day after surgery, the mice were sacrificed and perfused with normal saline and 4% paraformaldehyde. Then, the brains were fixed in 4% paraformaldehyde for 24 h and cut into 3 μm coronal paraffin sections.

### Hematoxylin–eosin (H&E) staining

The rehydrated sections were immersed in hematoxylin (G1004, Servicebio, Wuhan, China) and eosin Y (G1001, Servicebio) for 5 min and 20 s, respectively. Then, the slices were sealed with neutral balsam, and the pre-hematoma regions were captured by an M2 imager microscope (Carl Zeiss, Oberkochen, Germany).

### Immunofluorescent staining

After rehydrated, the slices were immersed in citric acid solution (PH 6.0) and heated with a microwave for antigen repair. Then, 3% H_2_O_2_ and 3% bovine serum albumin + 0.02% triton-X100 were used for blocking sequentially. Next, primary antibodies were incubated at 4 ℃ for 14 h. Then, a horseradish peroxidase (HRP)-conjugated secondary antibody was incubated, followed by a 10-min visualization using a tyramide signal amplification system (AFIHC023, AiFang Biological, Changsha, China) [37]. The primary antibodies included rabbit anti-ionized calcium binding adaptor molecule 1 (Iba1, microglia/macrophage marker, 1:2000, 17198, Cell Signaling Technology, Danvers, MA, USA), rabbit anti-proliferating cell nuclear antigen (PCNA, proliferating cell marker, 1:1000, 13110, Cell Signaling Technology), and rabbit anti-TNFα (1:200, YT4689, ImmunoWay, Plano, TX, USA).

### Data analysis

Statistical analyses were performed by one-way ANOVA followed by Dunnett’s test using SPSS (version 26, IBM Corp., Armonk, NY, USA). A *P* < 0.05 was regarded as statistically significant. Data were expressed as mean ± standard diversion.

## Results and discussion

### Microbial and hepatic biotransformation enhances the bioavailability and BBB permeability of ASIV

**Fig. 2:**
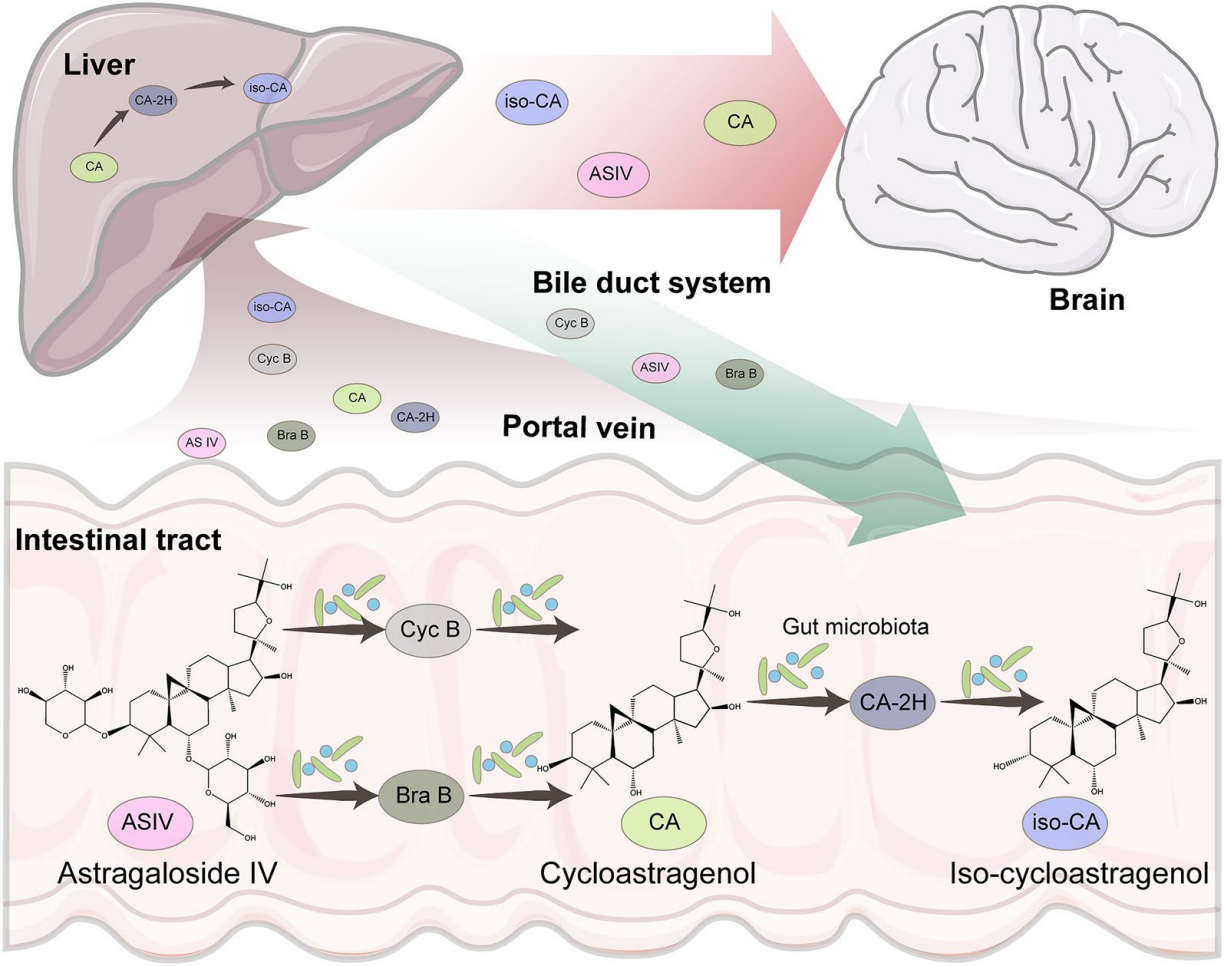
Microbial and hepatic biotransformation of ASIV. ASIV is transformed by gut microbiota to form Cyc B, Bra B, CA, CA-2H, and iso-CA. The microbiota-transformed products are infused into the liver. Then CA is partly converted into CA-2H and iso-CA. The bile duct system exerts Cyc B, Bra B, and ASIV. Only ASIV, CA, and iso-CA are detectable in the bloodstream. *ASIV* Astragaloside IV, *CA* cycloastragenol; *iso-CA* 3-*epi*-cycloastragenol, *Bra B* 6-*O*-β-d-glucopyranosyl, *Cyc B* 3-*O*-β-d-xylopyranosyl-cycloastragenol; *CA-2H* dehydrogenated to 20R, 24S-epoxy-6α, 16β, 25-tyihydroxy-9-cycloartan-3-one

To find out the potential effective compounds of ASIV, we analyzed the microbial and hepatic biotransformation by retrieving literature [21, 22]. As shown in Fig. 2, a little of ASIV was absorbed into the portal vein. The rest was mixed with the gut microbiota. Then, the xylose or glucose moiety was removed to form 6-*O*-β-d-glucopyranosyl (Bra B) and 3-*O*-β-d-xylopyranosyl-cycloastragenol (Cyc B), respectively. Next, the microbiota sequentially degraded the products into CA, 20R, 24S-epoxy-6α, 16β, 25-tyihydroxy-9-cycloartan-3-one (CA-2H), and iso-CA. The six compounds mentioned above were first infused into the liver through the portal vein and then underwent intrahepatic conversion and biliary exertion. As a result, only ASIV, CA, and iso-CA were detected in the bloodstream.

The ADMET prediction indicated that the transformed metabolites of ASIV processed lower molecular weight (MW), easier synthetic accessibility, and higher GI absorption, bioavailability, and DL (Table 1). These results were in agreement with the previous study indicating much higher OB of CA (25.7%) than ASIV (2.2–3.7%) in rats [22, 38]. Therefore, CA and iso-CA detected in the blood may be the additional active compounds of ASIV. Moreover, while ASIV was difficult to permeate the BBB, its microbial-transformed products, CA and iso-CA, could enter the brain parenchyma (Table 1). Taken together, ASIV, CA, and iso-CA were the candidate effective compounds of ASIV. Moreover, CA and iso-CA, other than ASIV itself, seemed to be the major effectors of ASIV in targeting CNS where BBB exists. Therefore, it is necessary to add the biotransformation analysis before the network pharmacology scheme to explore the therapeutic mechanism of ASIV in treating ICH.

**Table 1:** The predicted ADME properties, drug-likeness, pharmacokinetic, and physicochemical properties of Astragaloside IV and its derivates

Compounds	MW	Lipophilicity	Water solubility	GI absorption	BBB permeability	Bioavailability score	Synthetic accessibility	DL
ASIV	784.97	4.22	Moderate	Low	–	0.17	9.73	No
CA	490.72	3.97	Moderate	High	+	0.55	7.28	Yes
Iso-CA	490.72	4.28	Moderate	High	+	0.55	7.28	Yes

*MW* molecular weight, *GI* gastrointestinal, *BBB* blood–brain barrier, *DL* drug-likeness, *ASIV* astragaloside IV, *CA* iso-CA CA: cycloastragenol, *iso-CA* 3-epi-cycloastragenol

### Microbial and hepatic biotransformation increases the potential targets of ASIV in treating ICH

**Fig. 3:**
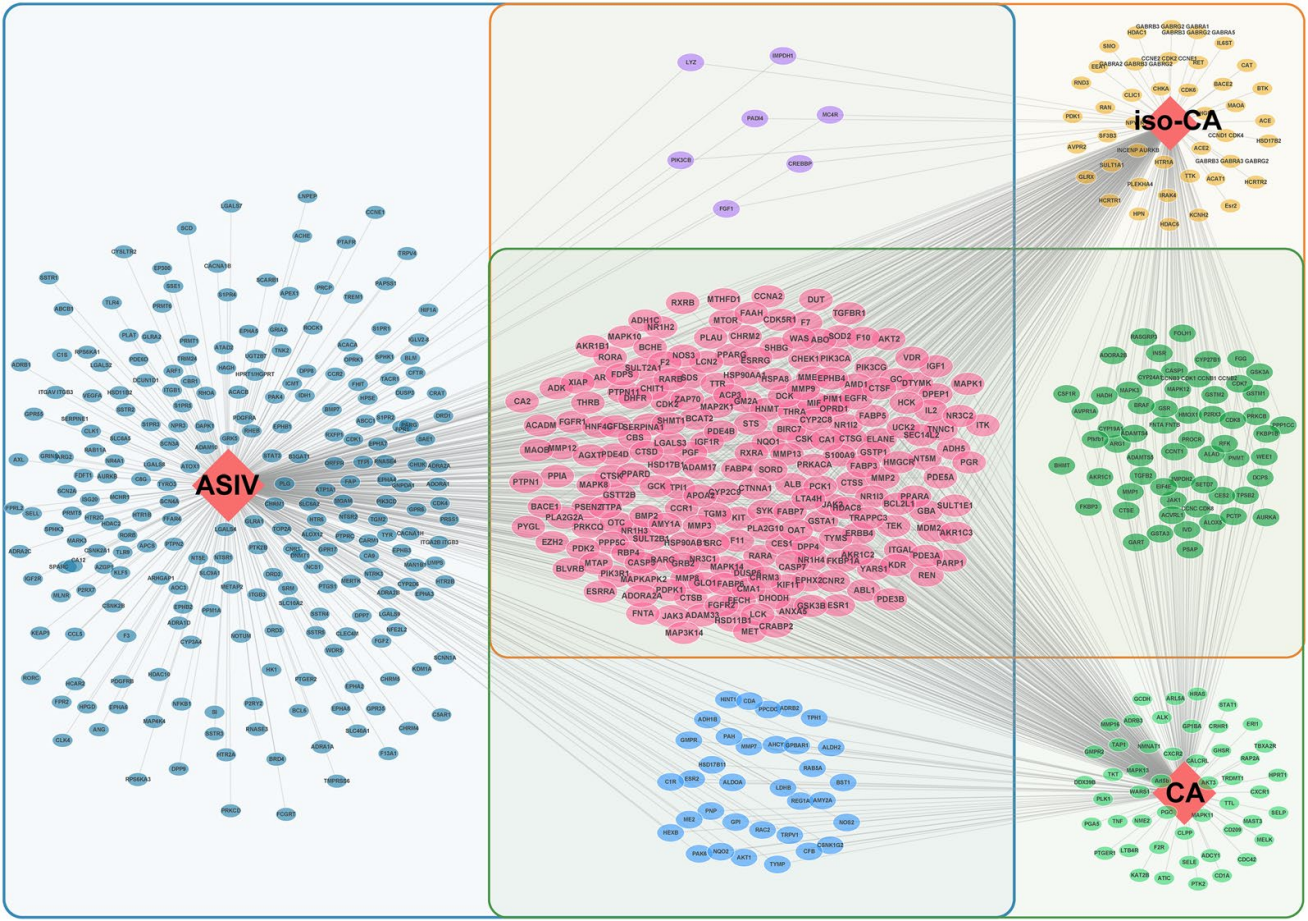
The potential targets of ASIV before and after microbial and hepatic biotransformation. There are 373 and 524 targets of ASIV before and after biotransformation, respectively. Two hundred and fifteen targets are common for ASIV, CA, and iso-CA (purple circles). The biotransformation results in 150 additional targets. *ASIV* Astragaloside IV, *CA* cycloastragenol, *iso-CA* 3-epi-cycloastragenol

To evaluate the advantage of our new strategy in discovering new targets, we screened the potential targets of ASIV before and after biotransformation. SwissTargetPrediction, SEA, and PharmMapper databases were applied. These tools work differently to predict targets: SwissTargetPrediction and SEA prediction rely on the topologic similarity to the known ligands [30, 39], and PharmMapper is based on a pharmacophore mapping approach [32]. As a result, a total of 373, 339, and 335 proteins were targeted by ASIV, CA, and iso-CA, respectively (Fig. 3). Among them, as many as 215 targets were common for ASIV, CA, and iso-CA. It is probably because of their similar backbones and functional groups. The results suggest that biotransformation enhances the abilities of ASIV to affect its targets. Moreover, 150 proteins were additionally targeted by CA or (and) iso-CA but not by ASIV. It implies that microbial and hepatic biotransformation add new effectors to ASIV functions.

**Fig. 4:**
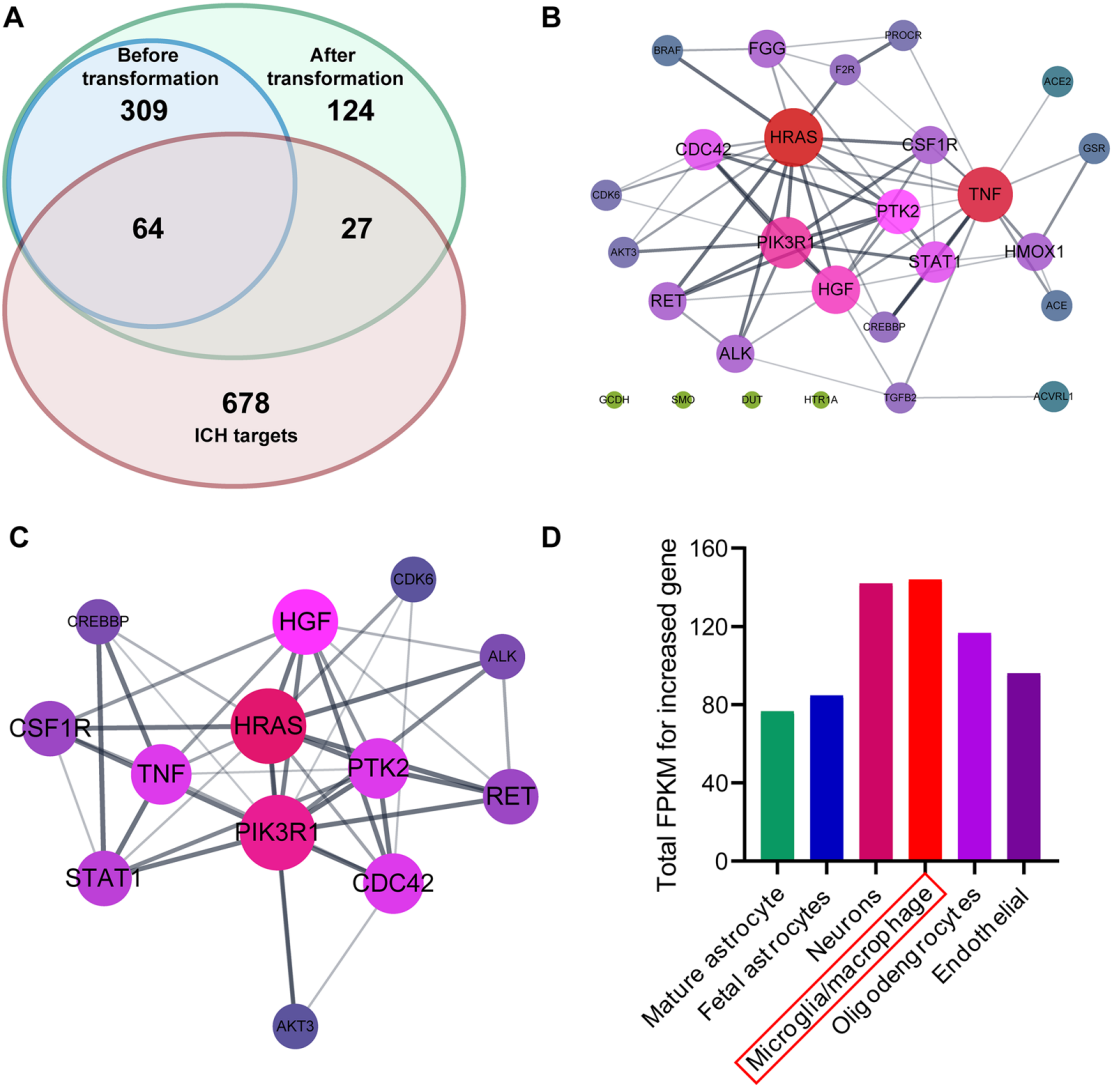
The biotransformation-added targets of ASIV on ICH. **A** Twenty-seven additional genes overlap with ICH targets. **B** The PPI network of the 27 additional targets. **C** The distribution analysis of 27 additional targets suggests that the biotransformation products primarily act on microglia/macrophages. **D** The core subnetwork of 27 additional targets shows the central role of HRAS, PIK3R1, PTK2, CDC42, CSF1R, HGF, STAT1, and TNF

To address the importance of microbial and hepatic biotransformation in ASIV treatment for ICH. The ICH targets were screened out and intersected with the compound targets before and after transformation. As displayed in the Venn diagram, 64 ICH-related genes were also targeted by ASIV. After biotransformation, the ASIV-associated ICH targets increased by 27 (Fig. 4A). The pre-transformation ASIV targets-ICH PPI network contained 64 nodes and 663 edges (Additional file 1: Fig. S1A). And the active compound-targets-ICH network included 91 nodes and much more edges (1192) than ASIV alone (Additional file 1: Fig. S1B). The additional targets-ICH network comprised 27 nodes and 63 edges (Fig. 4B). The core clusters resulting from MCODE included 13 nodes. The associations of these targets with ICH and the corresponding ingredients are displayed in Table 2. Among them, the central nodes predominantly included genes associated with cell migration (PTK2, CDC42, CSF1R, HGF), proliferation (PIK3R1, HGF, CSF1R), and inflammation (STAT1, TNF). (Fig. 4C). Then, to uncover the targeted cell type of the additional genes after biotransformation, we mapped the 27 additional genes to Brain RNA-Seq, a brain transcriptome database. The transformation-added targets are primarily expressed in microglia/macrophage (Fig. 4D).

**Table 2:** The associations of the core genes to ICH and the herbal products

Target	Compound	Relations to herbal products			Relations to ICH			
		Swiss^a^ target prediction	Pharm^b^ mapper	S^c^EA	Gene^d^ cards	OM IM	DisGe^e^NET	GAD
AKT3	CA	26/0	–	–	19.046	–	–	–
ALK	CA	42/0	–	–	14.515	–	–	–
CDC42	CA	–	2.797	–	10.492	–	–	–
CDK6	Iso-CA	–	2.499	–	12.655	–	–	–
CREBBP	Iso-CA	7/0	–	–	12.566	–	–	–
CSF1R	CA	50/0	–	–	18.709	–	–	–
	Iso-CA	63/0	–	–				
HGF	Iso-CA	–	2.996	–	10.667	–	–	–
HRAS	CA	–	2.348	–	19.016	–	–	–
PIK3R1	CA	–	3.081	–	14.633	–	–	–
	Iso-CA	–	4.418	–				
PTK2	CA	–	2.348	–	11.550	–	–	–
RET	Iso-CA	15/0	–	–	10.987	Yes	0.1	–
STAT1	CA	–	2.567	–	11.556	–	–	–
TNF	CA	4/0	–	–	30.716	–	–	Yes

^a^The number of hit known actives (3D/2D) in the SwissTargetPrediction database

^b^The fit scores in the PharmMapper database

^c^The Max Tc in the SEA database

^d^The relevance scores in the GeneCards database

^e^The disease-gene association scores in the DisGeNET database

**Fig. 5:**
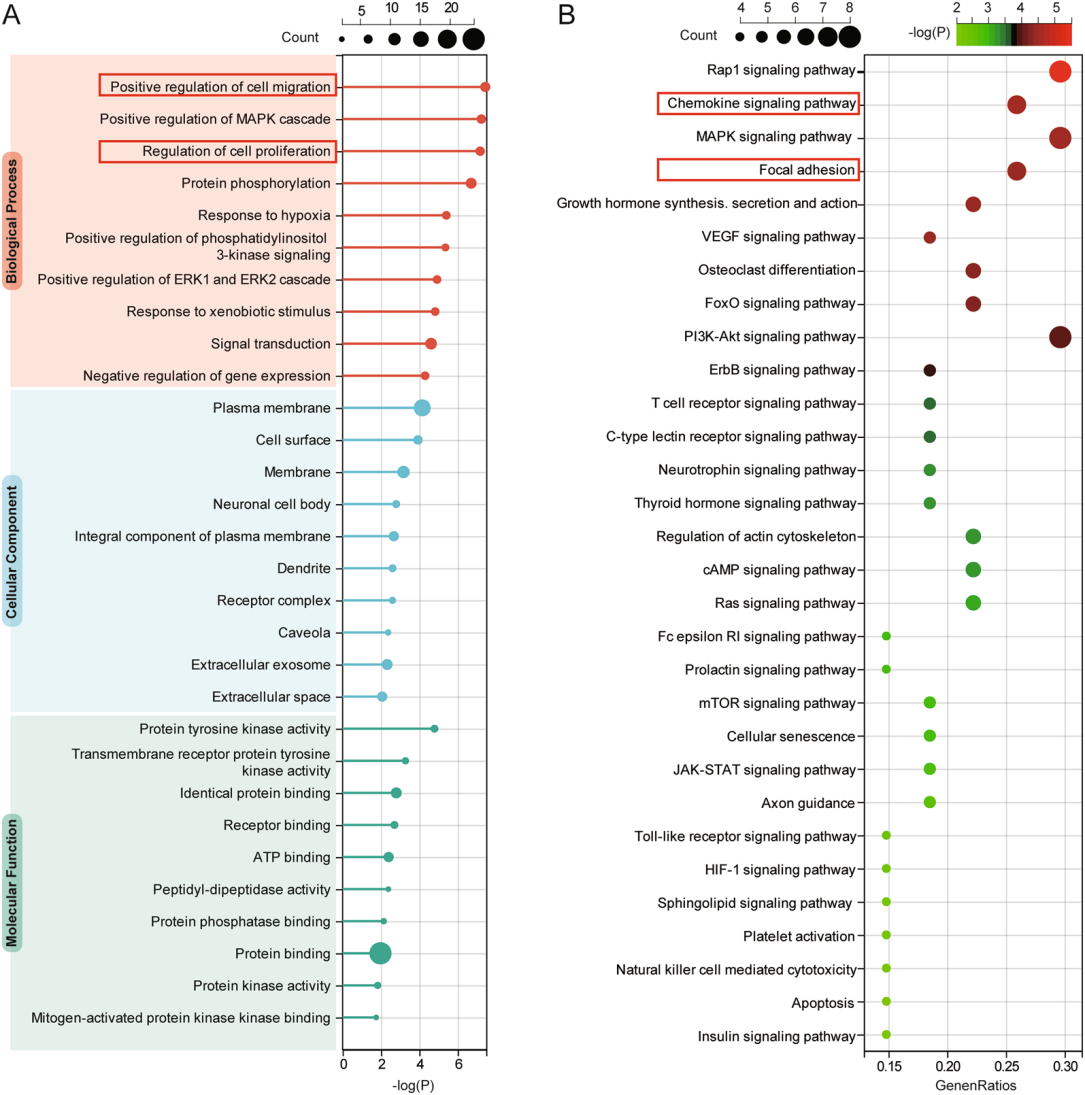
GO and KEGG analyses of the biotransformation-added targets. **A** The top 10 biological processes, cellular components, and molecular functions of ASIV enriched in GO analysis emphasize cell migration and proliferation. **B** The top 30 KEGG pathways indicate the essential roles of chemokines and focal adhesion on ASIV in treating ICH

Further functional enrichment analyses of the additional targets after ASIV biotransformation showed that most of the top enriched pathways were also relevant to cell migration (GO: positive regulation of cell migration; KEGG: Rap1 signaling pathway; KEGG: Focal adhesion), proliferation (GO: regulation of cell proliferation), and inflammation (GO: chemokine signaling pathway) (Fig. 5; Additional file 1: Table S1), which differed from the results of ASIV [17] (Additional file 1: Table S2).

Microglia is the resident macrophage in the central nervous system (CNS). It is the primary cell to regulate neuro-inflammation by producing cytokines like TNF-α. Besides, the activated microglia can proliferate and migrate. [33]. Though indispensable in CNS homeostasis, microglia and brain-infiltrating monocyte-derived macrophage are the instigators of secondary brain injury after ICH [34, 35]. Upon ICH, the perihematomal microglia/macrophage is activated as early as 1 h and 4 h in the collagenase- and auto-blood-induced ICH models, respectively [34]. In addition, microglia migrate to the lesions within 12 h [36]. Then, microglia/macrophage increases 8 folds in 3–5 days, exacerbating inflammation and secondary brain damage [34, 36]. Therefore, we focused on the biological processes directly related to microglia/macrophage activity. As a result, “GO: positive regulation of macrophage chemotaxis” and “GO: macrophage differentiation” were screened out, where CSF1R and PTK2, CDC42, and CSF1R hit (Additional file 1: Table S1). As predicted, CDC42, PTK2, and CSF1R were targeted by CA. In addition, CSF1R was also bound to iso-CA. In contrast, the biological processes “GO: positive regulation of macrophage chemotaxis” and “GO: macrophage differentiation” were not significantly enriched by the ASIV targets (Additional file 1: Table S2), nor were they reported by previous network pharmacology-based studies [17].

### Molecular docking and molecular dynamics simulation of ASIV derivatives and the additional targets.

We employed molecular docking to validate the additional effects of ASIV derivatives on ICH. As shown in Additional file 1: Fig. S2, CA formed 5 hydrogen bonds (H-bonds, blue dashed line) with CDC42, 5 H-bonds and 4 hydrophobic interactions (gray dashed line) with CSF1R, and 5 H-bonds and 6 hydrophobic interactions with PTK2, respectively. Besides, iso-CA (Blue-green sticks) was anchored into the active pocket of CSF1R via 4 H-bonds and 4 hydrophobic interactions. The lowest binding energy of CA-CDC42, CA-PTK2, CA-CSF1R, and iso-CA-CSF1R were − 7.5, − 7.3, − 7.9, and − 8.0 kcal/mol, respectively (Additional file 1: Fig. S2A–D; Table 3), indicating considerable binding affinities of the complexes.

**Table 3:** Results of molecular docking

Compounds	Targets	Binding energy (kcal/mol)	Hydrogen bonds	Hydrophobic interactions
CA	CDC42 (4js0)	− 7.5	Asp11, Asp11, Ser88, Asn92, Gln116	–
CA	PTK2 (6i8z)	− 7.3	Lys485, Leu486, Ser555, Lys-561	Tyr415, Leu449, Lys485, Ile487, Ile487, Glu500
CA	CSF1R (7mfc)	− 7.9	Asp670, Arg801, Arg801, Asp806, Asn808	Leu672, Arg782, Phe797, Arg801
Iso-CA	CSF1R (7mfc)	− 8.0	Asp670, Arg801, Arg801, Asn808	Leu672, Arg782, Phe797, Arg801

**Fig. 6:**
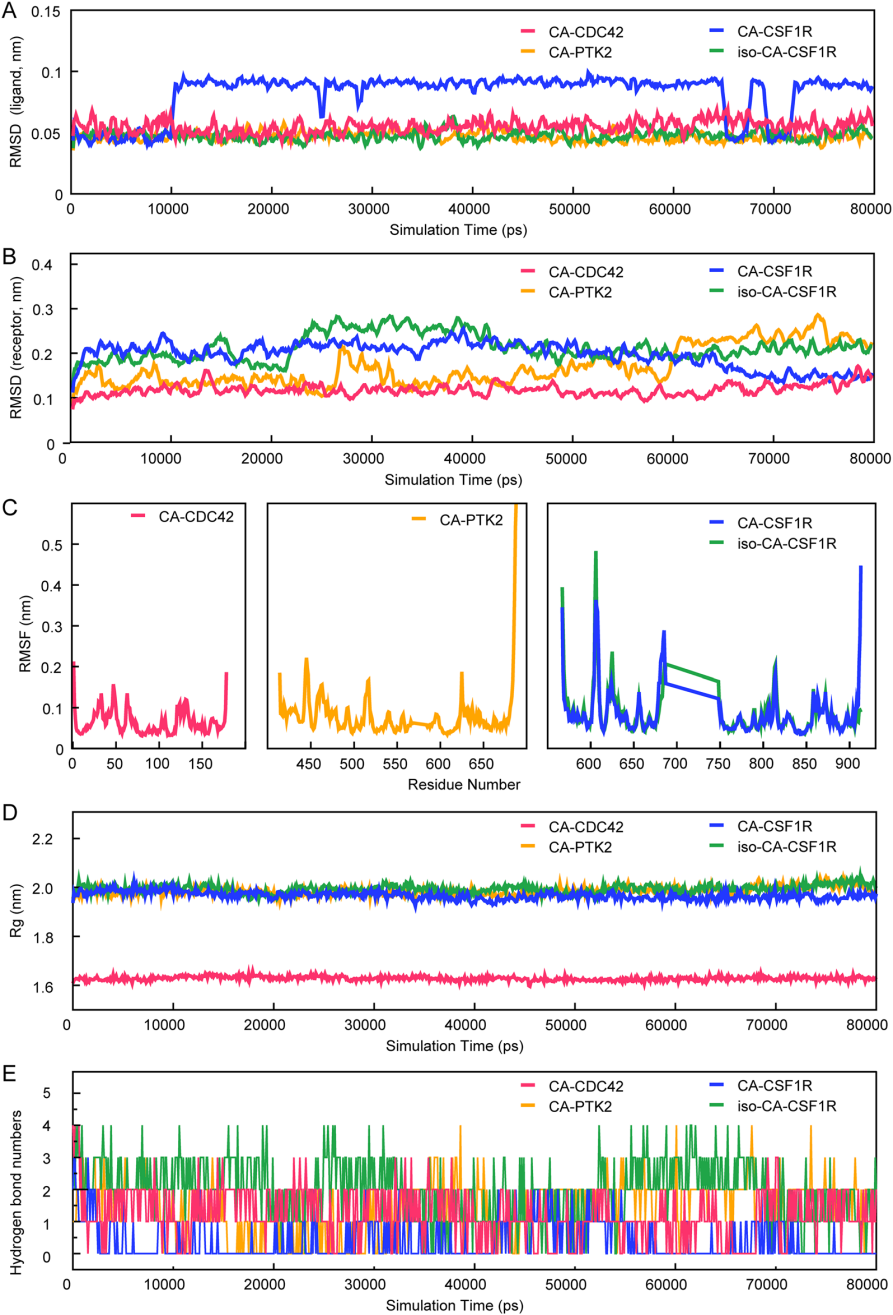
The dynamic stability of the ligand-target complexes. **A** The RMSD of ligands in compound-targets complexes show limited fluctuations in CA-CDC42, CA-PTK2, and iso-CA-CSF1R complexes but relatively large volatility in CA-CSF1R. **B** The RMSDs of receptors in compound-targets complexes show limited fluctuations in CA-CDC42, CA-PTK2, CA-CSF1R, and iso-CA-CSF1R complexes. **C** The RMSFs indicate that the residue-specific fluctuations of receptors are also stable, especially for the CA-CDC42 complex. **D** The Rgs of the four complexes are less fluctuated

**Fig. 7:**
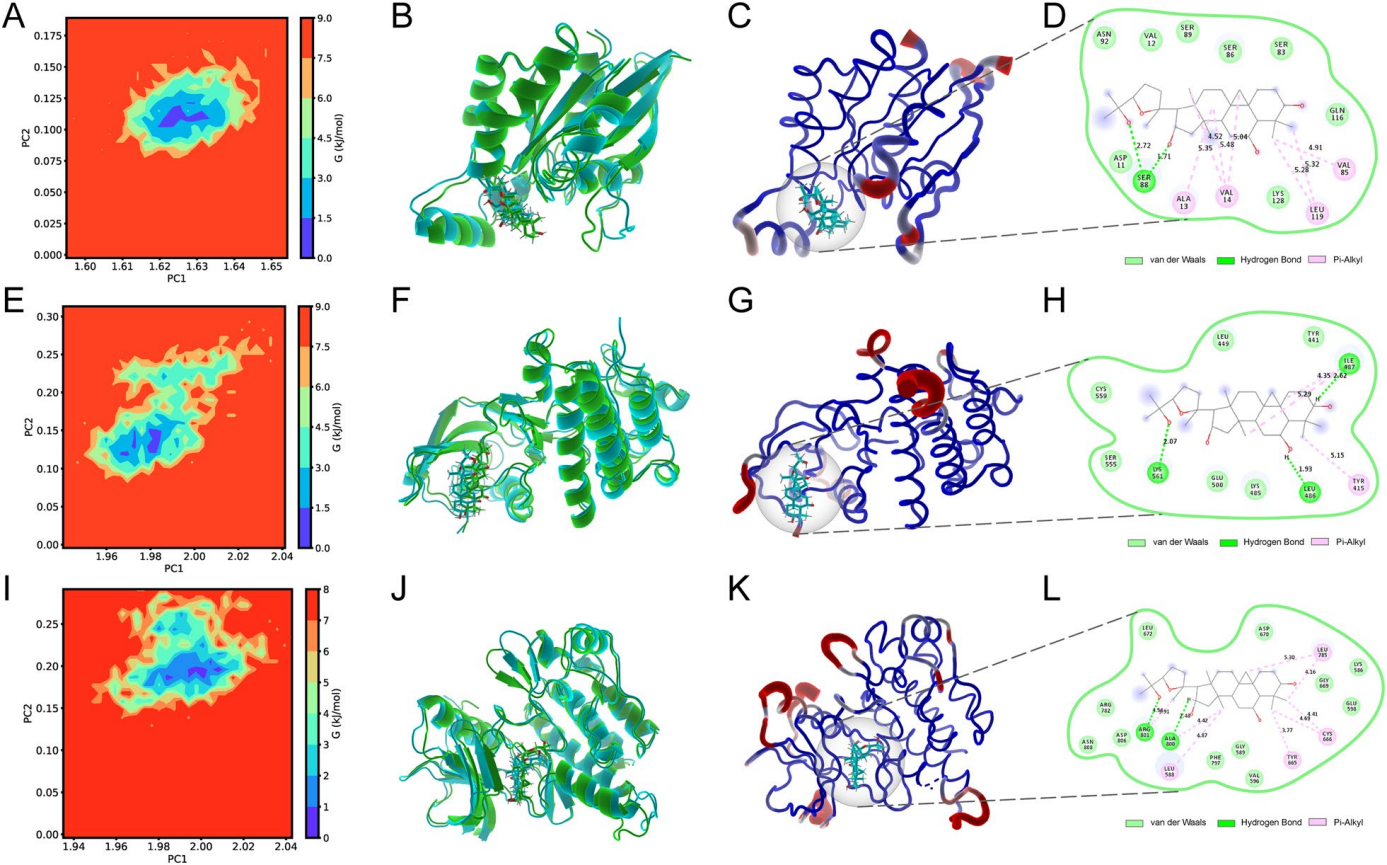
Exploration of the conformations with the lowest free binding energy. **A** The free energy landscape of CA-CDC42 during the 80-ns molecular dynamics simulation. 2D graph projects on the first two principal components (PC1 + PC2). Blue spots indicate the energy minima. **B** The overlapped graph of CA-CDC42 before (green) and after (blue) molecular dynamics simulation. **C** The low-energy conformation of CA-CDC42 is selected according to the free energy landscape. **D** The binding model of CA-CDC42 complex. Light green represents van der Waals, dark green represents hydrogen bonds, and pink represents hydrophobic interactions. **E** The free energy landscape of CA-PTK2 during the 80-ns molecular dynamics simulation. **F** The overlapped graph of CA-CDC42 before (green) and after (blue) molecular dynamics simulation. **G** The low-energy conformation of CA-PTK2 is selected according to the free energy landscape. **H** The binding model of CA-PTK2 complex. **I** The free energy landscape of iso-CA-CSF1R during the 80-ns molecular dynamics simulation. **J** The overlapped graph of iso-CA-CSF1R before (green) and after (blue) molecular dynamics simulation. **K** The low-energy conformation of iso-CA-CSF1R is selected according to the free energy landscape. **L** The binding model of the iso-CA-CSF1R complex. **M** The free energy landscape of CA-CSF1R

Molecular docking calculates the most stable conformations of the ligand-target complexes statically. However, whether the putative interactions are dynamically stable in a virtual environment remains unknown. To address this, a molecular dynamics simulation was performed. During the simulation, the atmosphere was set at physiological conditions, including ionic strength, temperature, and pressure [40]. In this method, root mean square deviation (RMSD) reflects the dynamic variations in the conformational stability of backbone molecules to the initial state. Root mean square fluctuation (RMSF) represents the stability of specific residues in the receptors. And the radius of gyration (Rg) evaluates the structural compactness of complex biological systems [41]. The results indicated that during the 80-ns simulation. The RMSD and Rg of the CA-CDC42, CA-PTK2, and iso-CA-CSF1R complexes were relatively smooth with limited fluctuation (Fig. 6A–D). Moreover, the RMSFs were low, especially for CA-CDC42, which suggested satisfactory stability of the above complexes. In these complexes, the ligands bound to the active pockets of their corresponding receptors and formed 0–4 hydrogen bonds during the 80-ns simulations (Fig. 6E). The estimated lowest binding free energy of CA-CDC42, CA-PTK2, and iso-CA-CSF1R were − 87.675 kJ/mol, − 64.234 kJ/mol, and − 84.796 kJ/mol, respectively. Although the lowest binding free energy of CA-CSF1R was relatively low (− 67.632 kJ/mol), the overall dynamic performance of the CA-CSF1R complex was poor, as suggested by the sizeable RMSD fluctuation (Fig. 7A), unfixed binding pocket (Additional file 1: Fig. S3B), and limited H-bonds (Fig. 6E). The free energy landscape and the conformations with the lowest energy were displayed (Fig. 7A–M; Additional file 1: Fig. S3A–B). Taken together, CA binds CDC42 and PTK2, and iso-CA binds CSF1R firmly. The results indicate that the transformed products may target CDC42, PTK2, and CSF1R directly.

CSF1R is the receptor of macrophage-CSF (M-CSF) and interleukin (IL)-34, whose activation contributes to microglia/macrophage survival, proliferation, chemotaxis, and proinflammation [42–45]. Once phosphorylated, CSF1R activates downstream factors, CDC42 included, to rapidly reorganize the actin cytoskeleton and focal adhesions, resulting in microglia/macrophage movement [44, 46]. In addition, CSF1R also triggers proliferating signaling pathways, like PI3K/Akt, JNK, and ERK1/2 pathways [44]. CSF1R blockage reduces microglia number and inflammation [45, 47, 48]. PTK2, or focal adhesion kinase (FAK), is one of the primary controllers of cell mobility by regulating cytoskeletal or cell adhesion site dynamics [49, 50]. Moreover, it can also activate CDC42 [51]. A previous study suggests that PTK2 inhibition impairs microglia migration [52]. Thus, we speculate that forming CA-CDC42, CA-PTK2, and iso-CA-CSF1R complexes can inhibit microglia/macrophage migration, proliferation, and chemokine secretion after ICH. ASIV may not fulfill these effects alone, because CDC42, PTK2, and CSF1R are not its targets.

### The orally administrated ASIV inhibits microglia/macrophage migration, proliferation, and chemokine secretion

**Fig. 8:**
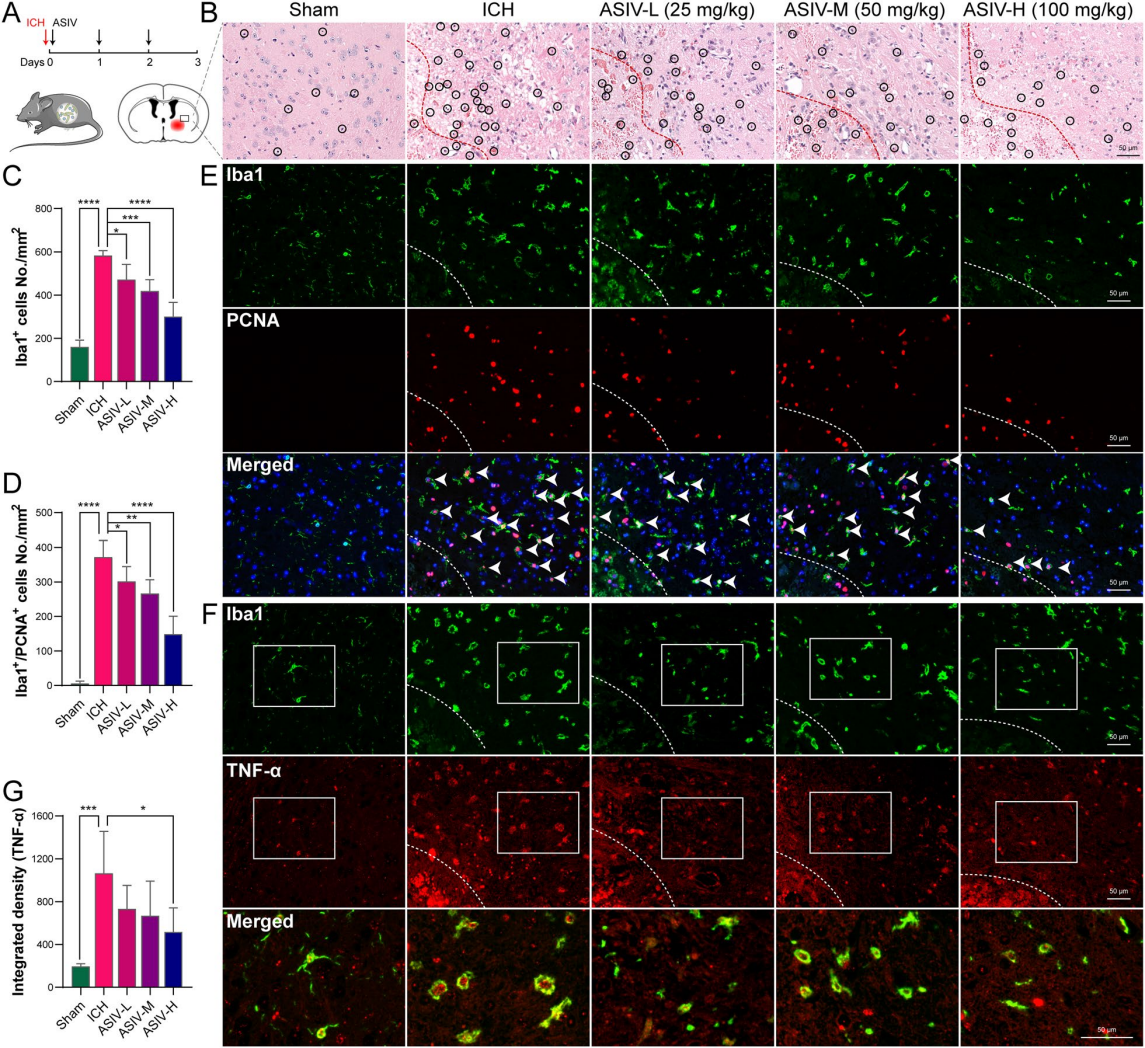
The effects of orally administrated ASIV on microglia/macrophage proliferation, migration, and chemokine secretion after ICH. **A** Flow chart of the animal experiments. **B** H&E staining suggests that ASIV alleviates brain disorganization and inflammatory cell infiltration. **C** The statistic graph of Iba1 immunofluorescent suggests that ASIV dose-dependently reduces the number of perihematomal microglia/macrophages. **D** The statistic graph of Iba1 and PCNA double staining suggests that ASIV reduces the number of proliferating microglia/macrophage (green cells encircles red nuclei). **E** The representative images of Iba1 (green) and PCNA (red) immunofluorescent. **F** The representative images of Iba1 (green) and TNF-α (red) double staining indicate that TNF-α is predominantly expressed by microglia/macrophage. **G** The statistical graph of TNF-α immunofluorescent suggests that a high dose of ASIV declines TNF-α production. Dashed line: hematoma (**B**, **E**, **F**). Data are expressed as mean ± SD, n = 5. *P < 0.05, **P < 0.01, ***P < 0.001, ****P < 0.0001. Scale bar = 50 μm

To further verify the putative interactions, we validated the additional targets and biological processes of transformed ASIV derivatives on ICH. Thus, we orally administrated ASIV to mice after ICH induction (Fig. 8A). As previously reported, ASIV delivered in this way can be transformed by gut microbiota and liver sequentially, resulting in considerable concentrations of CA and iso-CA in the bloodstream [21, 22]. Then, microglia/macrophage migration and proliferation were evaluated. As predicted by the bioinformatic analyses above, these processes might be promoted by CDC42, PTK2, and CSF1R while suppressed by the transformed products, CA or iso-CA.

H&E staining showed that in the ICH group, the cells were disorganized with a looser extracellular matrix. Inflammatory cells were under infiltration (Fig. 8B, black circles). After treatment, the perihematomal disorganization and inflammation were reduced (Fig. 8B). Immunofluorescent staining suggested that the total microglia/macrophage and proliferating microglia/macrophage were significantly diminished in the ASIV-treated groups, especially for the high dose. The results implied that ASIV alleviates microglia/macrophage migration and proliferation (Fig. 8C–E). Moreover, a high dose of ASIV decreased the perihematomal TNF-α, the pro-inflammatory factor mainly secreted by microglia/macrophages in the brain (Fig. 8F–G).

**Fig. 9:**
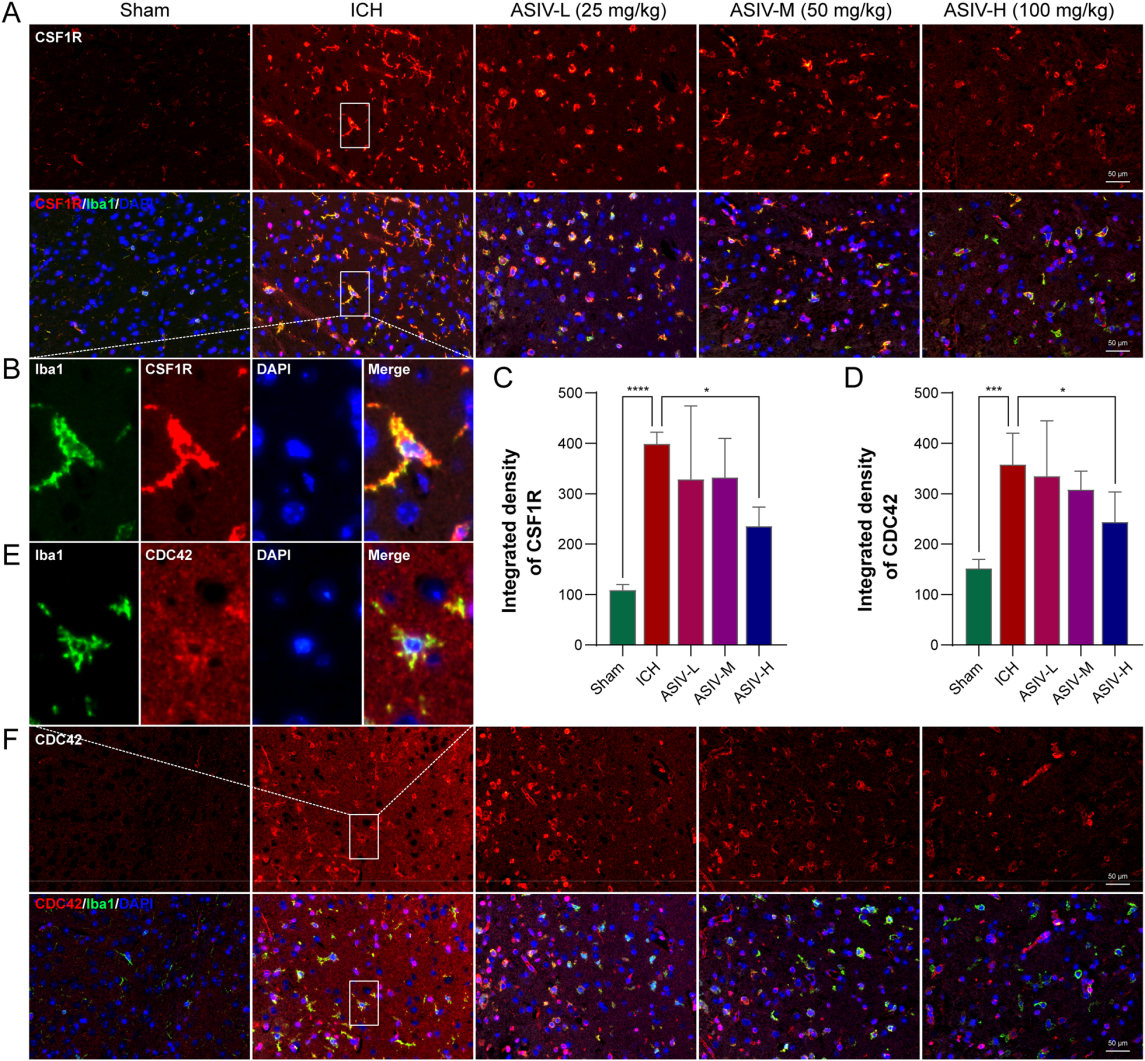
The effects of orally administrated ASIV on CDC42 and CSF1R after ICH. **A** The representative double-staining images of Iba1 (green) and CSF1R (red) show that ASIV decreases CSF1R expression. **B** The enlarged images show that CSF1R is mainly expressed in microglia/macrophage. **C** The statistical graph indicates that ASIV suppresses the expression of CSF1R. **D** The statistical graph indicates that ASIV suppresses the expression of CDC42. **E** The enlarged images show that CDC42 (red) is highly expressed on Iba1-positive microglia/macrophage (green). **F** The representative double-staining images of Iba1 and CDC42 show ASIV decreases CDC42 expression. Data are expressed as mean ± SD, n = 5. *P < 0.05, ***P < 0.001, ****P < 0.0001. Scale bar = 50 μm

As indicated by double immunofluorescent staining, the proliferation- and migration-promoting targets, CDC42 and CSF1R, were primarily expressed on Iba1-positive microglia/macrophage (Fig. 9B, E). They were significantly upregulated in the peri-hematoma brains after ICH. CDC42 and CSF1R were compromised after ASIV treatment (Fig. 9). It suggested that the binding of the transformed products on CDC42 and CSF1R potentially leads to their dysfunction and degradation. Moreover, the ASIV-reduced CDC42 and CSF1R might be responsible for the inhibited microglia/macrophage migration.

Our present results are supported by previous research, which demonstrates that intraperitoneally injection of ASIV or CA shifts microglia from a proinflammatory type to an anti-inflammatory one after brain ischemia [20, 23, 24]. In addition, a previous study applies a classic network pharmacology scheme to investigate the therapeutic mechanism of ASIV on ICH. It reports that orally administrated ASIV downregulates the levels of the master pro-inflammatory transcript factor, nuclear factor kappa-light-chain-enhancer of activated B cells (NF-κB), in the post-ICH brain [17]. In comparison, our new strategy additionally suggests that ASIV can inhibit microglia/macrophage proliferation and migration after microbial and hepatic biotransformation.

### CA but not ASIV suppresses microglia proliferation and migration in vitro

**Fig. 10:**
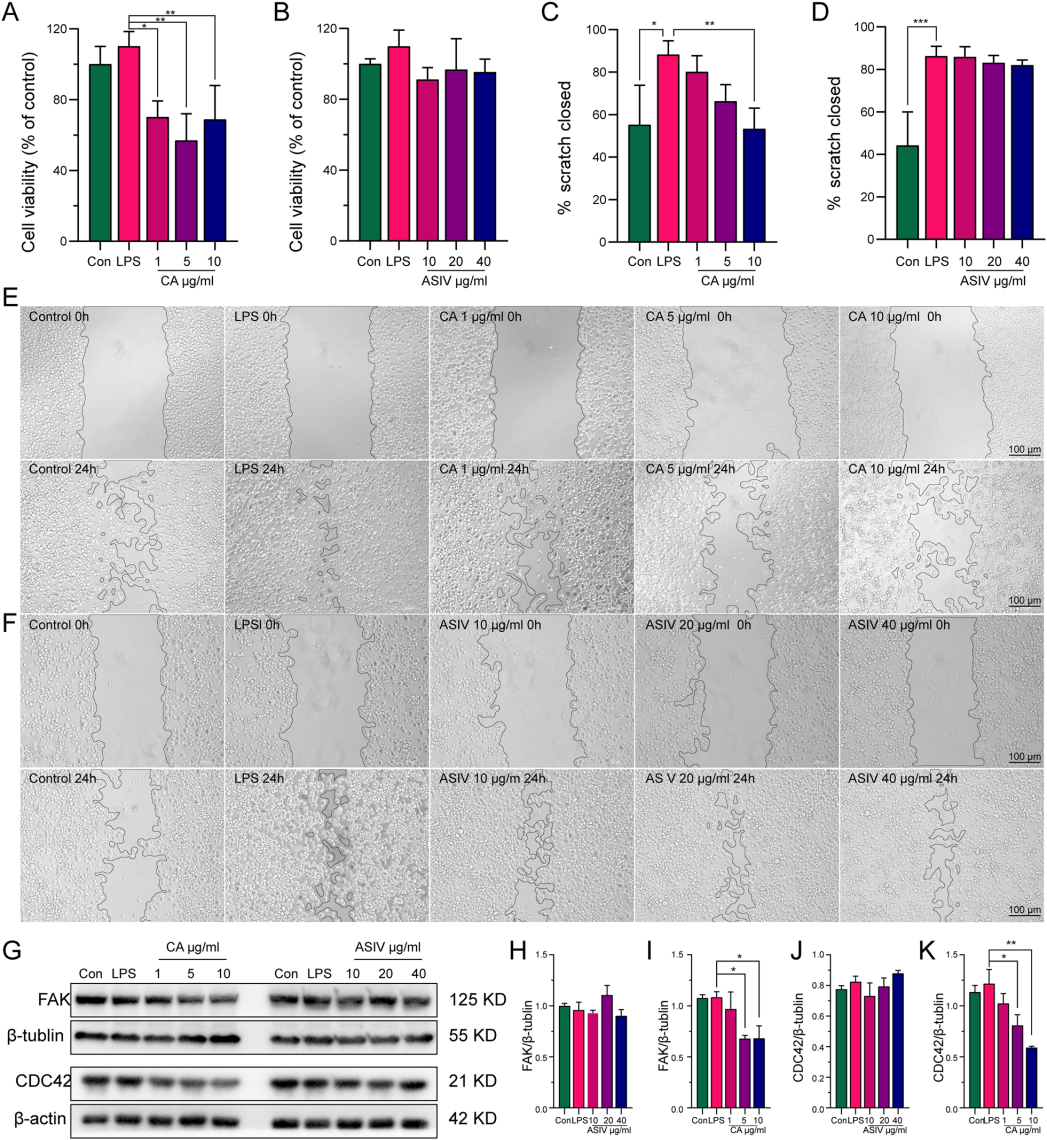
CA suppresses BV2 proliferation and migration, but ASIV does not. **A** CCK-8 shows that CA inhibits BV2 proliferation. **B** CCK-8 shows that ASIV doesn’t affect BV2 proliferation. **C** Scratch assay suggests that CA inhibits BV2 migration. **D** Scratch assay suggests that CA doesn’t inhibit BV2 migration. **E** Representative image of the scratch assay for CA. **F** Representative image of the scratch assay for ASIV. **G** Representative images of WB show that CA decreases FAK and CDC42 expressions. **H** The statistical graph shows that ASIV doesn’t affect the FAK level. **I** The statistical graph shows that CA decreases FAK expression. **J** The statistical graph shows that ASIV doesn’t affect CDC42 expression. **K** The statistical graph shows that CA suppresses the CDC42 level. Data are expressed as mean ± SD, n = 3. *P < 0.05, **P < 0.01, ***P < 0.001. Scale bar = 100 μm (**E**–**F**)

The in vivo study indicated that ASIV inhibited microglia/macrophage proliferation and migration after gut microbiota and liver biotransformation. However, the results could not distinguish the effects of the absorbed ASIV from those of the transformed products. Thus, we treated the microglia cell line (BV2) with ASIV or CA, one of the commercially available transformed products. The concentrations of ASIV and CA were determined according to the in vitro toxicity assay (Additional file 1: Fig. S4). Under LPS stimulation, CA but not ASIV suppressed BV2 proliferation (Fig. 10A–B) and migration (Fig. 10C–F). In addition, CA also reduced the expression of CDC42 and FAK in a dose-dependent manner (Fig. 10G–K).

## Conclusion

This study highlights a novel strategy that adds microbial and hepatic biotransformation analyses to the network pharmacology program. The new approach uncovers that the post-biotransformation products possess higher bioavailability and BBB permeability than the original form. Moreover, microbial and hepatic biotransformation enhances the efficacy of low-OB herbal products on their intrinsic targets and adds novel targets and biological processes. The transformed products may be the primary active compound of low-OB herbal ingredients in treating neurological diseases. Based on this strategy, the study reveals that ASIV inhibited proliferation, migration, and chemokine secretion of microglia/macrophage in the brain after ICH by its microbial- and hepatic-transformed products, through binding CDC42, PTK2, and CSF1R. However, the putative direct ligand-target interactions need to be proved in the future. This new strategy may help to explore the therapeutic mechanism of low-OB herbal products and TCM more systemically and thoroughly.

## Supplementary Information

**Additional** **file** **1:** **Figure** **S1.** PPI network of AS IV targets for ICH treatment before and after microbial and hepatic biotransformation. (A) PPI of intersected targets of AS IV and ICH. (B) PPI of intersected targets of ICH and potential effective compound after biotransformation. **Figure** **S2.** Molecular docking of AS IV derivates and the additional targets after biotransformation. (A) CA (purple) formed 5 hydrogen bonds with CDC42. (B) CA formed 4 hydrogen bonds and 6 hydrophobic interactions with PTK2. (C) CA formed 5 hydrogen bonds and 4 hydrophobic interactions with CSF1R. (D) Iso-CA formed 4 hydrogen bonds s and 4 hydrophobic interactions with CSF1R. Blue dashed line: hydrogen bonds; Gray dashed line: hydrophobic interactions. **Figure** **S3.** Molecular dynamic simulation of iso-CA-CSF1R. (A) Free energy landscapes CA-CSF1R during 80 ns molecular dynamic simulation. 2D graphs projected on the first two principal components (PC1 + PC2). Blue spots indicate the energy minima. (B) Overlapped graph of CA-CSF1R before (green) and after (blue) molecular dynamic simulation. **Figure** **S4.** In vitro toxicity assay of CA and AS IV on BV2. (A) Cell viability of BV2 after CA treatment. (B) Cell viability of BV2 after AS IV treatment. Data are expressed as mean ± SD, n = 3. * P < 0.05, ** P < 0.01, compared with control group. **Table** **S1.** Functional enrichment results of the biotransformation-added targets. **Table** **S2**. Functional enrichment results of the overlapped targets of ICH and AS IV.

## Data Availability

The data generated in this study are available from the corresponding author upon request.
