# Supplementary material for: A novel microbial and hepatic biotransformation-integrated network pharmacology strategy explores the therapeutic mechanisms of bioactive herbal products in neurological diseases: the effects of Astragaloside IV on intracerebral hemorrhage as an example
Source: Chin Med. 2023 Apr 17;18:40. doi: 10.1186/s13020-023-00745-5 (PMC10108474; doi:10.1186/s13020-023-00745-5)
Supplement: Supplementary file 1 — Additional file 1: Figure S1. PPI network of AS IV targets for ICH treatment before and after microbial and hepatic biotransformation. (A) PPI of intersected targets of AS IV and ICH. (B) PPI of intersected targets of ICH and potential effective compound after biotransformation. Figure S2. Molecular docking of AS IV derivates and the additional targets after biotransformation. (A) CA (purple) formed 5 hydrogen bonds with CDC42. (B) CA formed 4 hydrogen bonds and 6 hydrophobic interactions with PTK2. (C) CA formed 5 hydrogen bonds and 4 hydrophobic interactions with CSF1R. (D) Iso-CA formed 4 hydrogen bonds s and 4 hydrophobic interactions with CSF1R. Blue dashed line: hydrogen bonds; Gray dashed line: hydrophobic interactions. Figure S3. Molecular dynamic simulation of iso-CA-CSF1R. (A) Free energy landscapes CA-CSF1R during 80 ns molecular dynamic simulation. 2D graphs projected on the first two principal components (PC1 + PC2). Blue spots indicate the energy minima. (B) Overlapped graph of CA-CSF1R before (green) and after (blue) molecular dynamic simulation. Figure S4. In vitro toxicity assay of CA and AS IV on BV2. (A) Cell viability of BV2 after CA treatment. (B) Cell viability of BV2 after AS IV treatment. Data are expressed as mean ± SD, n = 3. * P < 0.05, ** P < 0.01, compared with control group. Table S1. Functional enrichment results of the biotransformation-added targets. Table S2. Functional enrichment results of the overlapped targets of ICH and AS IV. [file 13020_2023_745_MOESM1_ESM.docx]

**Additional file 1**

**
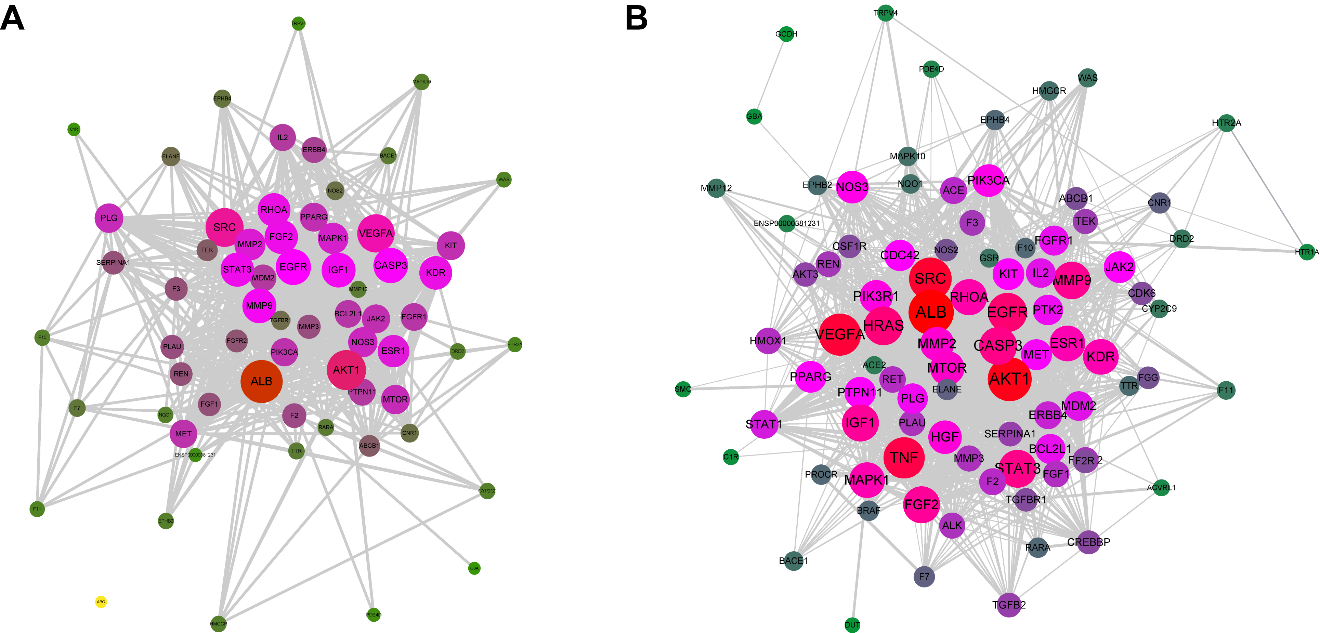
**

**Figure S1. PPI network of AS Ⅳ targets for ICH treatment before and after microbial and hepatic biotransformation.** (A) PPI of intersected targets of AS Ⅳ and ICH. (B) PPI of intersected targets of ICH and potential effective compound after biotransformation.


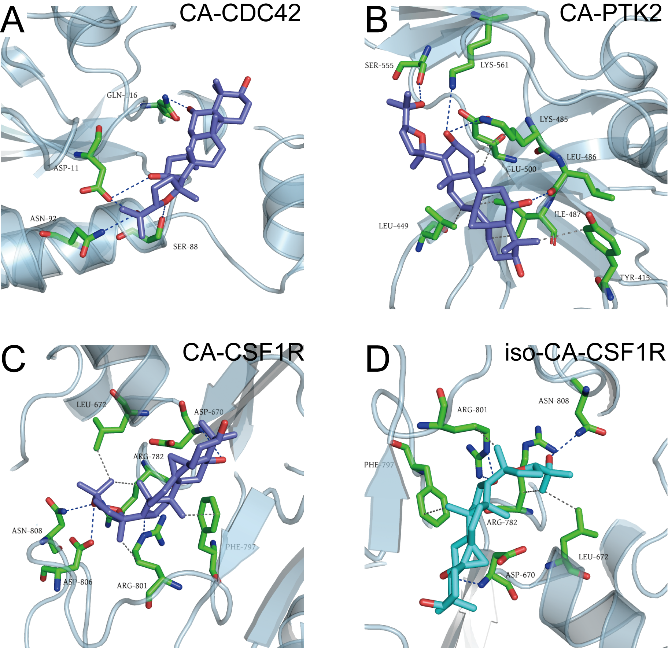


**Figure S2. Molecular docking** **of AS Ⅳ derivates and the additional targets after biotransformation.** (A) CA (purple) formed 5 hydrogen bonds with CDC42. (B) CA formed 4 hydrogen bonds and 6 hydrophobic interactions with PTK2. (C) CA formed 5 hydrogen bonds and 4 hydrophobic interactions with CSF1R. (D) Iso-CA formed 4 hydrogen bonds s and 4 hydrophobic interactions with CSF1R. Blue dashed line: hydrogen bonds; Gray dashed line: hydrophobic interactions.


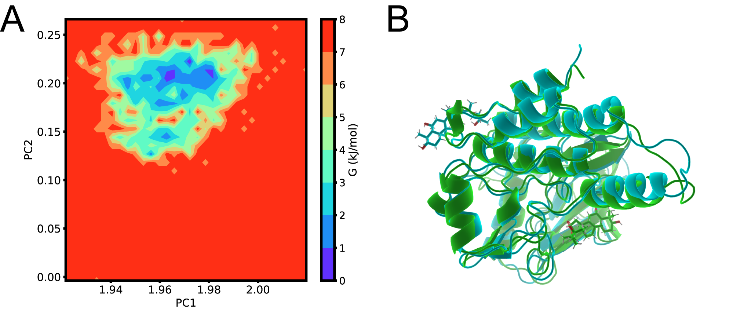


**Figure S3. Molecular dynamic simulation of iso-CA-CSF1R.** (A) Free energy landscapes CA-CSF1R during 80 ns molecular dynamic simulation. 2D graphs projected on the first two principal components (PC1 + PC2). Blue spots indicate the energy minima. (B) Overlapped graph of CA-CSF1R before (green) and after (blue) molecular dynamic simulation.


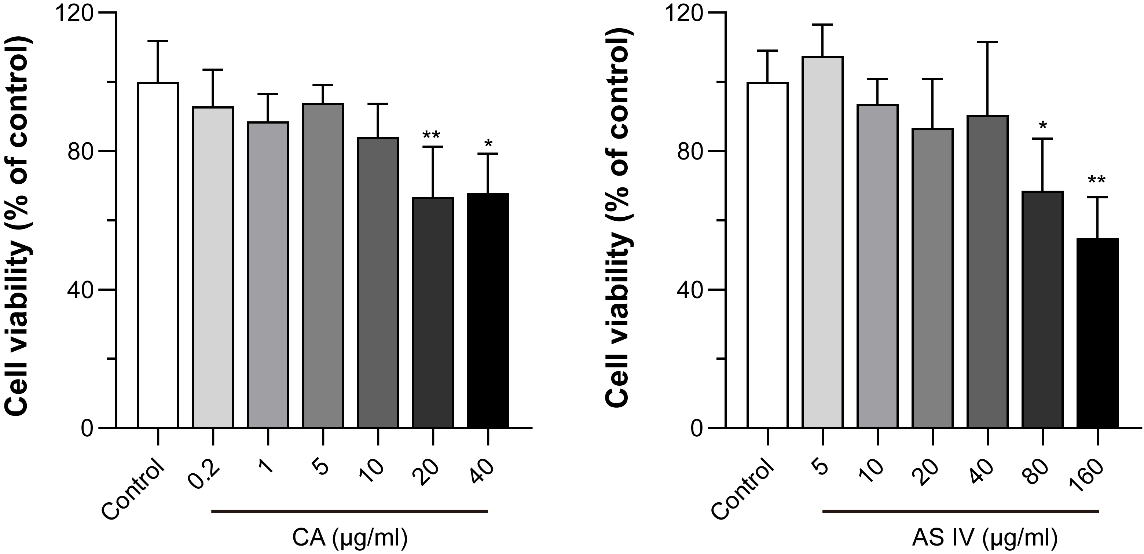


**Figure S4. In vitro toxicity assay of CA and AS IV on BV2.** (A) Cell viability of BV2 after CA treatment. (B) Cell viability of BV2 after AS IV treatment. Data are expressed as mean ± SD, n = 3. * P < 0.05, ** P < 0.01, compared with control group.

**Table S1. Functional enrichment results of the biotransformation-added targets.**

| **Category** | **Identifiers** | **Terms** | **Hit number** | **Hit ratios (%)** | **P value** | **Genes** |
| --- | --- | --- | --- | --- | --- | --- |
| GO-BP | GO:0030335 | Positive regulation of cell migration | 8 | 29.63 | 3.71E-08 | RET. CSF1R. SMO. HGF. F2R. PIK3R1. HRAS. PTK2 |
| GO-BP | GO:0043410 | Positive regulation of MAPK cascade | 7 | 25.93 | 5.92E-08 | CDC42. RET. ALK. HGF. F2R. HRAS. TNF |
| GO-BP | GO:0042127 | Regulation of cell proliferation | 7 | 25.93 | 6.85E-08 | ALK. ACE2. TGFB2. STAT1. BRAF. HRAS. PTK2 |
| GO-BP | GO:0006468 | Protein phosphorylation | 9 | 33.33 | 2.01E-07 | CDC42. RET. ACVRL1. TGFB2. CDK6. AKT3. BRAF. PIK3R1. PTK2 |
| GO-BP | GO:0001666 | Response to hypoxia | 6 | 22.22 | 3.95E-06 | ACVRL1. TGFB2. CREBBP. ACE. HMOX1. TNF |
| GO-BP | GO:0014068 | Positive regulation of Phosphatidylinositol 3-kinase signaling | 5 | 18.52 | 4.42E-06 | TGFB2. HGF. F2R. TNF. PTK2 |
| GO-BP | GO:0070374 | Positive regulation of ERK1 and ERK2 cascade | 6 | 22.22 | 1.21E-05 | CSF1R. F2R. FGG. BRAF. HRAS. TNF |
| GO-BP | GO:0009410 | Response to xenobiotic stimulus | 6 | 22.22 | 1.51E-05 | RET. TGFB2. ACE. STAT1. HMOX1. TNF |
| GO-BP | GO:0007165 | Signal transduction | 10 | 37.04 | 2.47E-05 | RET. ACVRL1. ALK. CSF1R. CREBBP. STAT1. AKT3. BRAF. PIK3R1. HRAS |
| GO-BP | GO:0010629 | Negative regulation of gene expression | 6 | 22.22 | 5.07E-05 | ACVRL1. TGFB2. ACE. SMO. HRAS. TNF |
| GO-BP | GO:0001934 | Positive regulation of protein phosphorylation | 5 | 18.52 | 0.000168 | CSF1R. HGF. HRAS. TNF. PTK2 |
| GO-BP | GO:0019229 | Regulation of vasoconstriction | 3 | 11.11 | 0.000326 | ACE2. ACE. HTR1A |
| GO-BP | GO:0008285 | Negative regulation of cell proliferation | 6 | 22.22 | 0.00034 | ACVRL1. CSF1R. TGFB2. CDK6. F2R. HRAS |
| GO-BP | GO:0043066 | Negative regulation of apoptotic process | 6 | 22.22 | 0.000603 | CSF1R. SMO. HGF. BRAF. PIK3R1. PTK2 |
| GO-BP | GO:1902042 | Negative regulation of extrinsic apoptotic signaling pathway via death domain receptors | 3 | 11.11 | 0.000645 | HGF. FGG. HMOX1 |
| GO-BP | GO:0048873 | Homeostasis of number of cells within a tissue | 3 | 11.11 | 0.000645 | SMO. AKT3. F2R |
| GO-BP | GO:0000165 | MAPK cascade | 4 | 14.81 | 0.000662 | RET. BRAF. HRAS. TNF |
| GO-BP | GO:0008284 | Positive regulation of cell proliferation | 6 | 22.22 | 0.000738 | CSF1R. TGFB2. F2R. HTR1A. HRAS. PTK2 |
| GO-BP | GO:0007169 | Transmembrane receptor protein tyrosine kinase signaling pathway | 4 | 14.81 | 0.00074 | RET. ALK. CSF1R. PTK2 |
| GO-BP | GO:0045944 | Positive regulation of transcription from RNA polymerase II promoter 8 | 8 | 29.63 | 0.000829 | ACVRL1. CREBBP. SMO. STAT1. HGF. PIK3R1. HRAS. TNF |
| GO-BP | GO:0045907 | Positive regulation of vasoconstriction | 3 | 11.11 | 0.00119 | ACE. F2R. FGG |
| GO-BP | GO:0043524 | Negative regulation of neuron apoptotic process | 4 | 14.81 | 0.001217 | F2R. HMOX1. BRAF. HRAS |
| GO-BP | GO:0001937 | Negative regulation of endothelial cell proliferation | 3 | 11.11 | 0.001253 | ACVRL1. STAT1. TNF |
| GO-BP | GO:0045766 | Positive regulation of angiogenesis | 4 | 14.81 | 0.001355 | ACVRL1. HGF. AKT3. HMOX1 |
| GO-BP | GO:0030316 | Osteoclast differentiation | 3 | 11.11 | 0.001384 | CSF1R. PIK3R1. TNF |
| GO-BP | GO:0061098 | Positive regulation of protein tyrosine kinase activity | 3 | 11.11 | 0.001593 | ALK. CSF1R. ACE |
| GO-BP | GO:1900026 | Positive regulation of substrate adhesion-dependent cell spreading | 3 | 11.11 | 0.001593 | CDC42. FGG. BRAF |
| GO-BP | GO:0032722 | Positive regulation of chemokine production | 3 | 11.11 | 0.001666 | CSF1R. HMOX1. TNF |
| GO-BP | GO:0008630 | Intrinsic apoptotic signaling pathway in response to DNA damage | 3 | 11.11 | 0.002053 | HMOX1. PIK3R1. TNF |
| GO-BP | GO:0045893 | Positive regulation of transcription. DNA-templated | 6 | 22.22 | 0.002512 | RET. ACVRL1. CREBBP. STAT1. F2R. TNF |
| GO-BP | GO:0043525 | Positive regulation of neuron apoptotic process | 3 | 11.11 | 0.003238 | CDC42. TGFB2. TNF |
| GO-BP | GO:0048661 | Positive regulation of smooth muscle cell proliferation | 3 | 11.11 | 0.003981 | STAT1. HMOX1. TNF |
| GO-BP | GO:0010693 | Negative regulation of alkaline phosphatase activity | 2 | 7.41 | 0.004035 | TGFB2. TNF |
| GO-BP | GO:0033674 | Positive regulation of kinase activity | 3 | 11.11 | 0.004321 | RET. ALK. CSF1R |
| GO-BP | GO:0010628 | Positive regulation of gene expression | 5 | 18.52 | 0.004725 | RET. CDK6. SMO. BRAF. TNF |
| GO-BP | GO:0008217 | Regulation of blood pressure | 3 | 11.11 | 0.005166 | ACVRL1. ACE. HMOX1 |
| GO-BP | GO:0006919 | activation of cysteine-type endopeptidase activity involved in apoptotic process | 3 | 11.11 | 0.00542 | RET. F2R. TNF |
| GO-BP | GO:0042493 | Response to drug | 4 | 14.81 | 0.006946 | RET. TGFB2. STAT1. HMOX1 |
| GO-BP | GO:0046330 | Positive regulation of JNK cascade | 3 | 11.11 | 0.007655 | CDC42. HRAS. TNF |
| GO-BP | GO:0071346 | Cellular response to interferon-gamma | 3 | 11.11 | 0.007807 | CDC42. STAT1. TNF |
| GO-BP | GO:0003081 | Regulation of systemic arterial blood pressure by renin-angiotensin | 2 | 7.41 | 0.008053 | ACE2. ACE |
| GO-BP | GO:0007179 | Transforming growth factor beta receptor signaling pathway | 3 | 11.11 | 0.008114 | ACVRL1. TGFB2. PTK2 |
| GO-BP | GO:0045217 | Cell-cell junction maintenance | 2 | 7.41 | 0.010724 | CSF1R. F2R |
| GO-BP | GO:0048699 | Generation of neurons | 2 | 7.41 | 0.012057 | TGFB2. CDK6 |
| GO-BP | GO:0051897 | Positive regulation of protein kinase B signaling | 3 | 11.11 | 0.012963 | RET. TNF. PTK2 |
| GO-BP | GO:0002003 | Angiotensin maturation | 2 | 7.41 | 0.013388 | ACE2. ACE |
| GO-BP | GO:0072577 | Endothelial cell apoptotic process | 2 | 7.41 | 0.014717 | BRAF. TNF |
| GO-BP | GO:0018108 | Peptidyl-tyrosine phosphorylation | 3 | 11.11 | 0.015759 | RET. CSF1R. PTK2 |
| GO-BP | GO:0045793 | Positive regulation of cell size | 2 | 7.41 | 0.016044 | RET. AKT3 |
| GO-BP | GO:0060413 | Atrial septum morphogenesis | 2 | 7.41 | 0.016044 | TGFB2. SMO |
| GO-BP | GO:0060396 | Growth hormone receptor signaling pathway | 2 | 7.41 | 0.01737 | PIK3R1. PTK2 |
| GO-BP | GO:0003323 | Type B pancreatic cell development | 2 | 7.41 | 0.018694 | CDK6. SMO |
| GO-BP | GO:0033630 | Positive regulation of cell adhesion mediated by integrin | 2 | 7.41 | 0.021337 | RET. TGFB2 |
| GO-BP | GO:0021542 | Dentate gyrus development | 2 | 7.41 | 0.021337 | CDK6. SMO |
| GO-BP | GO:0048566 | Embryonic digestive tract development | 2 | 7.41 | 0.021337 | TGFB2. TNF |
| GO-BP | GO:0034116 | Positive regulation of heterotypic cell-cell adhesion | 2 | 7.41 | 0.021337 | FGG. TNF |
| GO-BP | GO:0048143 | Astrocyte activation | 2 | 7.41 | 0.022656 | SMO. TNF |
| GO-BP | GO:0046325 | Negative regulation of glucose import | 2 | 7.41 | 0.022656 | ACE. TNF |
| GO-BP | GO:0050995 | Negative regulation of lipid catabolic process | 2 | 7.41 | 0.022656 | ALK. TNF |
| GO-BP | GO:0003203 | Endocardial cushion morphogenesis | 2 | 7.41 | 0.023974 | ACVRL1. TGFB2 |
| GO-BP | GO:0046777 | Protein autophosphorylation | 3 | 11.11 | 0.024508 | ALK. CSF1R. PTK2 |
| GO-BP | GO:0007411 | Axon guidance | 3 | 11.11 | 0.025013 | RET. CSF1R. PTK2 |
| GO-BP | GO:0010759 | Positive regulation of macrophage chemotaxis | 2 | 7.41 | 0.025289 | CSF1R. PTK2 |
| GO-BP | GO:2000573 | Positive regulation of DNA biosynthetic process | 2 | 7.41 | 0.025289 | HGF. TNF |
| GO-BP | GO:2000251 | Positive regulation of actin cytoskeleton reorganization | 2 | 7.41 | 0.025289 | CDC42. HRAS |
| GO-BP | GO:0060047 | Heart contraction | 2 | 7.41 | 0.026603 | CDC42. ACE |
| GO-BP | GO:0043123 | Positive regulation of I-kappaB kinase/NF-kappaB signaling | 3 | 11.11 | 0.027339 | F2R. HMOX1. TNF |
| GO-BP | GO:0060218 | Hematopoietic stem cell differentiation | 2 | 7.41 | 0.027915 | ACE. CDK6 |
| GO-BP | GO:0002053 | Positive regulation of mesenchymal cell proliferation | 2 | 7.41 | 0.030534 | SMO. STAT1 |
| GO-BP | GO:0009792 | Embryo development ending in birth or egg hatching | 2 | 7.41 | 0.030534 | TGFB2. ACE |
| GO-BP | GO:0030225 | Macrophage differentiation | 2 | 7.41 | 0.033147 | CDC42. CSF1R |
| GO-BP | GO:0007275 | Multicellular organism development | 3 | 11.11 | 0.034236 | RET. ALK. CSF1R |
| GO-BP | GO:0090303 | Positive regulation of wound healing | 2 | 7.41 | 0.035752 | HRAS. PTK2 |
| GO-BP | GO:0097746 | Regulation of blood vessel diameter | 2 | 7.41 | 0.037053 | ACE2. ACE |
| GO-BP | GO:0000122 | Negative regulation of transcription from RNA polymerase II promoter | 5 | 18.52 | 0.039084 | CREBBP. CDK6. SMO. STAT1. TNF |
| GO-BP | GO:0010592 | Positive regulation of lamellipodium assembly | 2 | 7.41 | 0.039648 | CDC42. PIK3R1 |
| GO-BP | GO:2000773 | Negative regulation of cellular senescence | 2 | 7.41 | 0.039648 | CDK6. AKT3 |
| GO-BP | GO:0031069 | Hair follicle morphogenesis | 2 | 7.41 | 0.040943 | TGFB2. SMO |
| GO-BP | GO:0043392 | Negative regulation of DNA binding | 2 | 7.41 | 0.042237 | SMO. HMOX1 |
| GO-BP | GO:0043552 | Positive regulation of phosphatidylinositol 3-kinase activity | 2 | 7.41 | 0.042237 | CDC42. PTK2 |
| GO-BP | GO:0045740 | Positive regulation of DNA replication | 2 | 7.41 | 0.042237 | CDC42. HRAS |
| GO-BP | GO:0051491 | Positive regulation of filopodium assembly | 2 | 7.41 | 0.042237 | CDC42. PIK3R1 |
| GO-BP | GO:0008015 | Blood circulation | 2 | 7.41 | 0.044819 | ACVRL1. STAT1 |
| GO-BP | GO:0001525 | Angiogenesis | 3 | 11.11 | 0.0449 | ACVRL1. HMOX1. PTK2 |
| GO-BP | GO:0090050 | Positive regulation of cell migration involved in sprouting angiogenesis | 2 | 7.41 | 0.046107 | AKT3. HMOX1 |
| GO-BP | GO:2000352 | Negative regulation of endothelial cell apoptotic process | 2 | 7.41 | 0.047394 | FGG. BRAF |
| GO-BP | GO:0048662 | Negative regulation of smooth muscle cell proliferation | 2 | 7.41 | 0.047394 | ACE2. HMOX1 |
| GO-BP | GO:0046427 | Positive regulation of JAK-STAT cascade | 2 | 7.41 | 0.048679 | F2R. TNF |
| GO-BP | GO:0016477 | Cell migration | 3 | 11.11 | 0.049803 | CDC42. TGFB2. PTK2 |
| GO-BP | GO:0001974 | Blood vessel remodeling | 2 | 7.41 | 0.049963 | ACVRL1. ACE |
| GO-BP | GO:0008219 | Cell death | 2 | 7.41 | 0.049963 | TGFB2. HMOX1 |
| GO-CC | GO:0005886 | Plasma membrane | 17 | 62.96 | 7.12E-05 | ALK. RET. ACVRL1. CSF1R. ACE. F2R. FGG. HTR1A. BRAF. PIK3R1. TNF. PTK2. CDC42. ACE2. PROCR. SMO. HRAS |
| GO-CC | GO:0009986 | Cell surface | 7 | 25.93 | 0.000119 | ACVRL1. PROCR. ACE2. CSF1R. F2R. FGG. TNF |
| GO-CC | GO:0016020 | Membrane | 11 | 40.74 | 0.000673 | CDC42. RET. ACVRL1. ACE2. ACE. HGF. AKT3. HMOX1. PIK3R1. HRAS. TNF |
| GO-CC | GO:0043025 | Neuronal cell body | 5 | 18.52 | 0.001616 | CDC42. RET. ACVRL1. TGFB2. TNF |
| GO-CC | GO:0005887 | Integral component of plasma membrane | 8 | 29.63 | 0.002141 | RET. ACVRL1. ALK. PROCR. CSF1R. F2R. HTR1A. TNF |
| GO-CC | GO:0030425 | Dendrite | 5 | 18.52 | 0.002501 | RET. ACVRL1. SMO. STAT1. HTR1A |
| GO-CC | GO:0043235 | Receptor complex | 4 | 14.81 | 0.00252 | RET. ACVRL1. ALK. CSF1R |
| GO-CC | GO:0005901 | Caveola | 3 | 11.11 | 0.004244 | SMO. F2R. HMOX1 |
| GO-CC | GO:0070062 | Extracellular exosome | 9 | 33.33 | 0.004749 | CDC42. ALK. PROCR. ACE2. DUT. ACE. SMO. GSR. FGG |
| GO-CC | GO:0005615 | Extracellular space | 8 | 29.63 | 0.008632 | PROCR. ACE2. TGFB2. ACE. HGF. FGG. HMOX1. TNF |
| GO-CC | GO:0032991 | Macromolecular complex | 5 | 18.52 | 0.011128 | CDC42. ALK. STAT1. PIK3R1. TNF |
| GO-CC | GO:0005576 | Extracellular region | 8 | 29.63 | 0.014168 | PROCR. ACE2. TGFB2. ACE. HGF. F2R. FGG. TNF |
| GO-CC | GO:0048471 | Perinuclear region of cytoplasm | 5 | 18.52 | 0.014742 | PROCR. STAT1. HMOX1. PIK3R1. HRAS |
| GO-CC | GO:0009897 | External side of plasma membrane | 4 | 14.81 | 0.019296 | ACE. GSR. FGG. TNF |
| GO-CC | GO:0045121 | Membrane raft | 3 | 11.11 | 0.040086 | RET. ACE2. TNF |
| GO-MF | GO:0004713 | Protein tyrosine kinase activity | 5 | 18.52 | 1.65E-05 | RET. ALK. CSF1R. BRAF. PTK2 |
| GO-MF | GO:0004714 | Transmembrane receptor protein tyrosine kinase activity | 4 | 14.81 | 0.000531 | RET. ALK. CSF1R. PTK2 |
| GO-MF | GO:0042802 | Identical protein binding | 9 | 33.33 | 0.001602 | CDC42. ALK. ACE2. STAT1. HGF. FGG. HMOX1. BRAF. TNF |
| GO-MF | GO:0005102 | Receptor binding | 5 | 18.52 | 0.002 | TGFB2. HGF. F2R. FGG. PTK2 |
| GO-MF | GO:0005524 | ATP binding | 8 | 29.63 | 0.003994 | RET. ACVRL1. ALK. CSF1R. CDK6. AKT3. BRAF. PTK2 |
| GO-MF | GO:0008241 | Peptidyl-dipeptidase activity | 2 | 7.41 | 0.004128 | ACE2. ACE |
| GO-MF | GO:0019903 | Protein phosphatase binding | 3 | 11.11 | 0.007232 | CSF1R. PIK3R1. PTK2 |
| GO-MF | GO:0005515 | Protein binding | 24 | 88.89 | 0.010663 | ALK. RET. ACVRL1. CSF1R. TGFB2. CREBBP. DUT. STAT1. HGF. F2R. FGG. HTR1A. BRAF. PIK3R1. TNF. PTK2. CDC42. ACE2. PROCR. CDK6. SMO. AKT3. HMOX1. HRAS |
| GO-MF | GO:0004672 | Protein kinase activity | 4 | 14.81 | 0.014842 | RET. AKT3. BRAF. PTK2 |
| GO-MF | GO:0031434 | Mitogen-activated protein kinase kinase binding | 2 | 7.41 | 0.017771 | ACE. BRAF |
| GO-MF | GO:0004180 | Carboxypeptidase activity | 2 | 7.41 | 0.029896 | ACE2. ACE |
| GO-MF | GO:0016301 | Kinase activity | 3 | 11.11 | 0.038441 | RET. ALK. PIK3R1 |
| GO-MF | GO:0005164 | Tumor necrosis factor receptor binding | 2 | 7.41 | 0.043199 | STAT1. TNF |
| KEGG | hsa04015 | Rap1 signaling pathway | 8 | 0.30 | 2.93E-06 | CDC42. CSF1R. HGF. AKT3. F2R. BRAF. PIK3R1. HRAS |
| KEGG | hsa04062 | Chemokine signaling pathway | 7 | 0.26 | 2.43E-05 | CDC42. STAT1. AKT3. BRAF. PIK3R1. HRAS. PTK2 |
| KEGG | hsa04010 | MAPK signaling pathway | 8 | 0.30 | 2.67E-05 | CDC42. CSF1R. TGFB2. HGF. AKT3. BRAF. HRAS. TNF |
| KEGG | hsa04510 | Focal adhesion | 7 | 0.26 | 3.15E-05 | CDC42. HGF. AKT3. BRAF. PIK3R1. HRAS. PTK2 |
| KEGG | hsa04935 | Growth hormone synthesis. secretion and action | 6 | 0.22 | 3.24E-05 | CREBBP. STAT1. AKT3. PIK3R1. HRAS. PTK2 |
| KEGG | hsa04370 | VEGF signaling pathway | 5 | 0.19 | 3.27E-05 | CDC42. AKT3. PIK3R1. HRAS. PTK2 |
| KEGG | hsa04380 | Osteoclast differentiation | 6 | 0.22 | 4.42E-05 | CSF1R. TGFB2. STAT1. AKT3. PIK3R1. TNF |
| KEGG | hsa04068 | FoxO signaling pathway | 6 | 0.22 | 4.94E-05 | TGFB2. CREBBP. AKT3. BRAF. PIK3R1. HRAS |
| KEGG | hsa04151 | PI3K-Akt signaling pathway | 8 | 0.30 | 8.74E-05 | CSF1R. CDK6. HGF. AKT3. F2R. PIK3R1. HRAS. PTK2 |
| KEGG | hsa04012 | ErbB signaling pathway | 5 | 0.19 | 0.000137 | AKT3. BRAF. PIK3R1. HRAS. PTK2 |
| KEGG | hsa04660 | T cell receptor signaling pathway | 5 | 0.19 | 0.000299 | CDC42. AKT3. PIK3R1. HRAS. TNF |
| KEGG | hsa04625 | C-type lectin receptor signaling pathway | 5 | 0.19 | 0.000299 | STAT1. AKT3. PIK3R1. HRAS. TNF |
| KEGG | hsa04722 | Neurotrophin signaling pathway | 5 | 0.19 | 0.000501 | CDC42. AKT3. BRAF. PIK3R1. HRAS |
| KEGG | hsa04919 | Thyroid hormone signaling pathway | 5 | 0.19 | 0.000533 | CREBBP. STAT1. AKT3. PIK3R1. HRAS |
| KEGG | hsa04810 | Regulation of actin cytoskeleton | 6 | 0.22 | 0.000539 | CDC42. F2R. BRAF. PIK3R1. HRAS. PTK2 |
| KEGG | hsa04024 | cAMP signaling pathway | 6 | 0.22 | 0.000574 | CREBBP. AKT3. F2R. HTR1A. BRAF. PIK3R1 |
| KEGG | hsa04014 | Ras signaling pathway | 6 | 0.22 | 0.000774 | CDC42. CSF1R. HGF. AKT3. PIK3R1. HRAS |
| KEGG | hsa04664 | Fc epsilon RI signaling pathway | 4 | 0.15 | 0.001253 | AKT3. PIK3R1. HRAS. TNF |
| KEGG | hsa04917 | Prolactin signaling pathway | 4 | 0.15 | 0.001363 | STAT1. AKT3. PIK3R1. HRAS |
| KEGG | hsa04150 | mTOR signaling pathway | 5 | 0.19 | 0.001382 | AKT3. BRAF. PIK3R1. HRAS. TNF |
| KEGG | hsa04218 | Cellular senescence | 5 | 0.19 | 0.001382 | TGFB2. CDK6. AKT3. PIK3R1. HRAS |
| KEGG | hsa04630 | JAK-STAT signaling pathway | 5 | 0.19 | 0.001588 | CREBBP. STAT1. AKT3. PIK3R1. HRAS |
| KEGG | hsa04360 | Axon guidance | 5 | 0.19 | 0.002433 | CDC42. SMO. PIK3R1. HRAS. PTK2 |
| KEGG | hsa04620 | Toll-like receptor signaling pathway | 4 | 0.15 | 0.00422 | STAT1. AKT3. PIK3R1. TNF |
| KEGG | hsa04066 | HIF-1 signaling pathway | 4 | 0.15 | 0.004814 | CREBBP. AKT3. HMOX1. PIK3R1 |
| KEGG | hsa04071 | Sphingolipid signaling pathway | 4 | 0.15 | 0.006148 | AKT3. PIK3R1. HRAS. TNF |
| KEGG | hsa04611 | Platelet activation | 4 | 0.15 | 0.00689 | AKT3. F2R. FGG. PIK3R1 |
| KEGG | hsa04650 | Natural killer cell mediated cytotoxicity | 4 | 0.15 | 0.007202 | BRAF. PIK3R1. HRAS. TNF |
| KEGG | hsa04210 | Apoptosis | 4 | 0.15 | 0.008883 | AKT3. PIK3R1. HRAS. TNF |
| KEGG | hsa04910 | Insulin signaling pathway | 4 | 0.15 | 0.009063 | AKT3. BRAF. PIK3R1. HRAS |
| KEGG | hsa04072 | Phospholipase D signaling pathway | 4 | 0.15 | 0.011184 | AKT3. F2R. PIK3R1. HRAS |
| KEGG | hsa04213 | Longevity regulating pathway - multiple species | 3 | 0.11 | 0.016404 | AKT3. PIK3R1. HRAS |
| KEGG | hsa04929 | GnRH secretion | 3 | 0.11 | 0.017421 | AKT3. PIK3R1. HRAS |
| KEGG | hsa04720 | Long-term potentiation | 3 | 0.11 | 0.018995 | CREBBP. BRAF. HRAS |
| KEGG | hsa04662 | B cell receptor signaling pathway | 3 | 0.11 | 0.027713 | AKT3. PIK3R1. HRAS |
| KEGG | hsa04610 | Complement and coagulation cascades | 3 | 0.11 | 0.029618 | PROCR. F2R. FGG |
| KEGG | hsa04211 | Longevity regulating pathway | 3 | 0.11 | 0.032238 | AKT3. PIK3R1. HRAS |
| KEGG | hsa04350 | TGF-beta signaling pathway | 3 | 0.11 | 0.035638 | TGFB2. CREBBP. TNF |
| KEGG | hsa04666 | Fc gamma R-mediated phagocytosis | 3 | 0.11 | 0.037742 | CDC42. AKT3. PIK3R1 |
| KEGG | hsa04914 | Progesterone-mediated oocyte maturation | 3 | 0.11 | 0.041353 | AKT3. BRAF. PIK3R1 |
| KEGG | hsa04931 | Insulin resistance | 3 | 0.11 | 0.045853 | AKT3. PIK3R1. TNF |
| KEGG | hsa04668 | TNF signaling pathway | 3 | 0.11 | 0.04895 | AKT3. PIK3R1. TNF |
| KEGG | hsa04725 | Cholinergic synapse | 3 | 0.11 | 0.049736 | AKT3. PIK3R1. HRAS |

**Table S2. Functional enrichment results of the overlapped targets of ICH and AS Ⅳ.**

| **Category** | **Identifiers** | **Terms** | **Hit number** | **Hit ratios (%)** | **P value** | **Genes** |
| --- | --- | --- | --- | --- | --- | --- |
| GO-BP | GO:0030335 | positive regulation of cell migration | 18 | 26.86567 | 1.1E-17 | F10, MMP2, STAT3, IGF1, FGF1, F3, MMP9, RHOA, EGFR, TGFBR1, VEGFA, F7, PLAU, KIT, KDR, MAPK1, EPHB2, JAK2 |
| GO-BP | GO:0051897 | positive regulation of protein kinase B signaling | 14 | 20.89552 | 4.22E-16 | F10, SRC, IGF1, FGF2, F3, EGFR, TGFBR1, MTOR, VEGFA, F7, PIK3CA, TEK, MET, FGFR1 |
| GO-BP | GO:0070374 | positive regulation of ERK1 and ERK2 cascade | 15 | 22.38806 | 3.29E-14 | SRC, PTPN11, HMGCR, IGF1, HTR2A, FGF1, FGF2, EGFR, VEGFA, ERBB4, TRPV4, KDR, TEK, DRD2, FGFR2 |
| GO-BP | GO:0033674 | positive regulation of kinase activity | 11 | 16.41791 | 5.6E-14 | ERBB4, KIT, KDR, TEK, EPHB2, MET, EPHB4, EGFR, IL2, FGFR2, FGFR1 |
| GO-BP | GO:0018108 | peptidyl-tyrosine phosphorylation | 13 | 19.40299 | 5.96E-14 | SRC, HTR2A, EGFR, ERBB4, KIT, KDR, TEK, EPHB2, JAK2, MET, EPHB4, FGFR2, FGFR1 |
| GO-BP | GO:0014068 | positive regulation of phosphatidylinositol 3-kinase signaling | 11 | 16.41791 | 2.14E-13 | ERBB4, SRC, KIT, KDR, TEK, IGF1, F2, JAK2, FGF2, FGFR1, VEGFA |
| GO-BP | GO:0043066 | negative regulation of apoptotic process | 18 | 26.86567 | 1.42E-12 | NQO1, SRC, IGF1, MMP9, EGFR, TGFBR1, IL2, MTOR, VEGFA, CBS, ERBB4, CASP3, ALB, MDM2, KDR, AKT1, TEK, BCL2L1 |
| GO-BP | GO:0048661 | positive regulation of smooth muscle cell proliferation | 10 | 14.92537 | 1.99E-12 | PIK3CA, MMP2, AKT1, HMGCR, IGF1, FGF2, EGFR, ELANE, FGFR2, MTOR |
| GO-BP | GO:0008284 | positive regulation of cell proliferation | 18 | 26.86567 | 2.9E-12 | IGF1, HTR2A, F2, FGF1, FGF2, EGFR, TGFBR1, IL2, VEGFA, ERBB4, KIT, MDM2, KDR, RARA, AKT1, FGFR2, FGFR1, BCL2L1 |
| GO-BP | GO:0046777 | protein autophosphorylation | 12 | 17.91045 | 2.49E-11 | ERBB4, SRC, KIT, KDR, AKT1, TEK, JAK2, EPHB4, EGFR, FGFR2, MTOR, FGFR1 |
| GO-BP | GO:0007169 | transmembrane receptor protein tyrosine kinase signaling pathway | 11 | 16.41791 | 2.6E-11 | ERBB4, SRC, KIT, KDR, TEK, EPHB2, MET, EPHB4, EGFR, FGFR2, FGFR1 |
| GO-BP | GO:0007275 | multicellular organism development | 12 | 17.91045 | 1.75E-10 | ERBB4, KIT, KDR, TEK, EPHB2, F2, FGF1, MET, EPHB4, EGFR, FGFR2, FGFR1 |
| GO-BP | GO:0043406 | positive regulation of MAP kinase activity | 9 | 13.43284 | 2.87E-10 | SRC, KIT, HTR2A, FGF1, FGF2, EGFR, ELANE, FGFR1, VEGFA |
| GO-BP | GO:0007596 | blood coagulation | 9 | 13.43284 | 6.23E-10 | F7, SERPINA1, F10, PLAU, F11, WAS, PLG, F2, F3 |
| GO-BP | GO:0010628 | positive regulation of gene expression | 15 | 22.38806 | 1.72E-09 | NOS3, STAT3, IGF1, FGF1, FGF2, F3, TGFBR1, MTOR, VEGFA, MDM2, RARA, AKT1, MAPK1, PPARG, EPHB2 |
| GO-BP | GO:0043410 | positive regulation of MAPK cascade | 10 | 14.92537 | 3.63E-09 | KIT, KDR, TEK, IGF1, JAK2, FGF2, TGFBR1, FGFR2, FGFR1, VEGFA |
| GO-BP | GO:0007165 | signal transduction | 21 | 31.34328 | 4.19E-09 | SRC, PDE4D, STAT3, IGF1, FGF1, FGF2, ESR1, EGFR, TGFBR1, MAPK10, TTR, PLAU, ERBB4, KIT, RARA, AKT1, MAPK1, PPARG, TEK, JAK2, MET |
| GO-BP | GO:0001938 | positive regulation of endothelial cell proliferation | 8 | 11.9403 | 7.83E-09 | KDR, AKT1, TEK, FGF2, F3, TGFBR1, MTOR, VEGFA |
| GO-BP | GO:0006508 | proteolysis | 13 | 19.40299 | 1.22E-08 | F10, MMP2, F11, MMP3, PLG, F2, MMP9, MMP12, BACE1, PLAU, CASP3, REN, ELANE |
| GO-BP | GO:0043536 | positive regulation of blood vessel endothelial cell migration | 7 | 10.44776 | 1.79E-08 | NOS3, KDR, AKT1, PLG, FGF2, FGFR1, VEGFA |
| GO-BP | GO:1904707 | positive regulation of vascular smooth muscle cell proliferation | 7 | 10.44776 | 3.21E-08 | SRC, MMP2, MDM2, IGF1, JAK2, FGF2, MMP9 |
| GO-BP | GO:0001934 | positive regulation of protein phosphorylation | 10 | 14.92537 | 3.88E-08 | ERBB4, KDR, AKT1, TEK, F2, FGF1, FGF2, MMP9, EGFR, VEGFA |
| GO-BP | GO:0042060 | wound healing | 8 | 11.9403 | 6.02E-08 | CASP3, IGF1, FGF1, FGF2, EGFR, TGFBR1, FGFR2, MTOR |
| GO-BP | GO:0001525 | angiogenesis | 10 | 14.92537 | 1.84E-07 | PIK3CA, NOS3, MMP2, KDR, TEK, EPHB2, FGF1, EPHB4, FGFR2, VEGFA |
| GO-BP | GO:0001666 | response to hypoxia | 9 | 13.43284 | 1.92E-07 | F7, NOS2, PLAU, CASP3, MMP2, TEK, DRD2, RHOA, VEGFA |
| GO-BP | GO:0007568 | aging | 9 | 13.43284 | 3.14E-07 | NQO1, CNR1, MMP2, STAT3, MAPK1, AKT1, HMGCR, HTR2A, FGF2 |
| GO-BP | GO:0045944 | positive regulation of transcription from RNA polymerase II promoter | 18 | 26.86567 | 3.97E-07 | STAT3, WAS, IGF1, FGF1, FGF2, ESR1, EGFR, IL2, VEGFA, MMP12, MDM2, RARA, AKT1, PPARG, JAK2, DRD2, MET, FGFR2 |
| GO-BP | GO:0045471 | response to ethanol | 8 | 11.9403 | 4.06E-07 | NQO1, CNR1, STAT3, RARA, HMGCR, RHOA, IL2, FGFR2 |
| GO-BP | GO:0022617 | extracellular matrix disassembly | 6 | 8.955224 | 4.92E-07 | MMP12, MMP2, MMP3, PLG, MMP9, ELANE |
| GO-BP | GO:0042493 | response to drug | 10 | 14.92537 | 6.55E-07 | NQO1, ABCB1, SRC, CASP3, STAT3, MDM2, PPARG, HTR2A, DRD2, RHOA |
| GO-BP | GO:0050673 | epithelial cell proliferation | 6 | 8.955224 | 6.79E-07 | KIT, KDR, DRD2, FGF1, EGFR, BCL2L1 |
| GO-BP | GO:0007173 | epidermal growth factor receptor signaling pathway | 6 | 8.955224 | 6.79E-07 | PIK3CA, ERBB4, SRC, AKT1, PTPN11, EGFR |
| GO-BP | GO:0002042 | cell migration involved in sprouting angiogenesis | 5 | 7.462687 | 7.13E-07 | KDR, AKT1, FGF2, EPHB4, VEGFA |
| GO-BP | GO:0030307 | positive regulation of cell growth | 7 | 10.44776 | 7.16E-07 | AKT1, F2, RHOA, EGFR, TGFBR1, IL2, MTOR |
| GO-BP | GO:0048013 | ephrin receptor signaling pathway | 6 | 8.955224 | 8.32E-07 | SRC, MMP2, PTPN11, EPHB2, MMP9, EPHB4 |
| GO-BP | GO:0050731 | positive regulation of peptidyl-tyrosine phosphorylation | 7 | 10.44776 | 8.66E-07 | SRC, PTPN11, IGF1, HTR2A, JAK2, MTOR, VEGFA |
| GO-BP | GO:0043491 | protein kinase B signaling | 6 | 8.955224 | 1.01E-06 | PIK3CA, KDR, AKT1, IGF1, DRD2, FGF2 |
| GO-BP | GO:0010629 | negative regulation of gene expression | 10 | 14.92537 | 1.02E-06 | PIK3CA, NOS2, STAT3, KDR, AKT1, PPARG, IGF1, ESR1, FGF2, VEGFA |
| GO-BP | GO:0032496 | response to lipopolysaccharide | 8 | 11.9403 | 1.07E-06 | NOS2, CNR1, NOS3, CASP3, REN, JAK2, ELANE, FGFR2 |
| GO-BP | GO:0045766 | positive regulation of angiogenesis | 8 | 11.9403 | 1.43E-06 | NOS3, STAT3, KDR, TEK, FGF1, FGF2, F3, VEGFA |
| GO-BP | GO:0010595 | positive regulation of endothelial cell migration | 6 | 8.955224 | 3.53E-06 | KDR, AKT1, TEK, FGF1, FGF2, VEGFA |
| GO-BP | GO:0043552 | positive regulation of phosphatidylinositol 3-kinase activity | 5 | 7.462687 | 4.17E-06 | ERBB4, SRC, KIT, TEK, FGF2 |
| GO-BP | GO:0043627 | response to estrogen | 6 | 8.955224 | 4.38E-06 | F7, MMP2, MAPK1, PPARG, TEK, ESR1 |
| GO-BP | GO:0010863 | positive regulation of phospholipase C activity | 4 | 5.970149 | 4.5E-06 | KIT, ESR1, FGF2, FGFR1 |
| GO-BP | GO:0050679 | positive regulation of epithelial cell proliferation | 6 | 8.955224 | 4.69E-06 | IGF1, FGF1, FGF2, EGFR, FGFR2, VEGFA |
| GO-BP | GO:0050927 | positive regulation of positive chemotaxis | 4 | 5.970149 | 8.21E-06 | F7, KDR, F3, VEGFA |
| GO-BP | GO:0034614 | cellular response to reactive oxygen species | 5 | 7.462687 | 8.42E-06 | MMP2, MAPK1, AKT1, MMP9, EGFR |
| GO-BP | GO:0071456 | cellular response to hypoxia | 7 | 10.44776 | 9.37E-06 | SRC, CBS, MDM2, AKT1, PPARG, MTOR, VEGFA |
| GO-BP | GO:0035094 | response to nicotine | 5 | 7.462687 | 1.04E-05 | CNR1, CASP3, MMP2, MAPK1, DRD2 |
| GO-BP | GO:0030324 | lung development | 6 | 8.955224 | 1.48E-05 | NOS3, FGF1, FGF2, EGFR, FGFR2, VEGFA |
| GO-BP | GO:0001541 | ovarian follicle development | 5 | 7.462687 | 1.53E-05 | MMP2, KIT, KDR, BCL2L1, VEGFA |
| GO-BP | GO:1904645 | response to beta-amyloid | 4 | 5.970149 | 1.69E-05 | MMP12, MMP2, MMP3, MMP9 |
| GO-BP | GO:0045893 | positive regulation of transcription, DNA-templated | 12 | 17.91045 | 3.17E-05 | ERBB4, SRC, STAT3, RARA, MAPK1, AKT1, PPARG, IGF1, ESR1, FGF2, EGFR, TGFBR1 |
| GO-BP | GO:0010507 | negative regulation of autophagy | 5 | 7.462687 | 3.46E-05 | STAT3, AKT1, MET, MTOR, BCL2L1 |
| GO-BP | GO:0032355 | response to estradiol | 6 | 8.955224 | 4.05E-05 | NQO1, F7, CASP3, STAT3, RARA, ESR1 |
| GO-BP | GO:0045165 | cell fate commitment | 5 | 7.462687 | 4.29E-05 | ERBB4, CASP3, KDR, PPARG, FGFR2 |
| GO-BP | GO:0042593 | glucose homeostasis | 6 | 8.955224 | 4.41E-05 | CNR1, TRPV4, STAT3, AKT1, PTPN11, PPARG |
| GO-BP | GO:0008543 | fibroblast growth factor receptor signaling pathway | 5 | 7.462687 | 4.6E-05 | PTPN11, FGF1, FGF2, FGFR2, FGFR1 |
| GO-BP | GO:0006809 | nitric oxide biosynthetic process | 4 | 5.970149 | 5.61E-05 | NQO1, NOS2, NOS3, AKT1 |
| GO-BP | GO:0009612 | response to mechanical stimulus | 5 | 7.462687 | 7.21E-05 | SRC, MMP2, TRPV4, PPARG, RHOA |
| GO-BP | GO:0007611 | learning or memory | 5 | 7.462687 | 7.65E-05 | SRC, CASP3, MAPK1, EPHB2, EGFR |
| GO-BP | GO:0030163 | protein catabolic process | 5 | 7.462687 | 8.6E-05 | CASP3, MMP2, AKT1, ELANE, MTOR |
| GO-BP | GO:0042531 | positive regulation of tyrosine phosphorylation of STAT protein | 5 | 7.462687 | 9.11E-05 | ERBB4, KIT, IGF1, JAK2, IL2 |
| GO-BP | GO:0007507 | heart development | 7 | 10.44776 | 0.000102 | ERBB4, CASP3, MMP2, PTPN11, PPARG, TEK, TGFBR1 |
| GO-BP | GO:2001243 | negative regulation of intrinsic apoptotic signaling pathway | 4 | 5.970149 | 0.000118 | SRC, AKT1, MMP9, BCL2L1 |
| GO-BP | GO:0051894 | positive regulation of focal adhesion assembly | 4 | 5.970149 | 0.000118 | KDR, PTPN11, TEK, VEGFA |
| GO-BP | GO:0043065 | positive regulation of apoptotic process | 8 | 11.9403 | 0.000126 | CNR1, SRC, CASP3, MMP2, AKT1, PPARG, MMP9, TGFBR1 |
| GO-BP | GO:0045776 | negative regulation of blood pressure | 4 | 5.970149 | 0.000131 | NOS2, CNR1, NOS3, DRD2 |
| GO-BP | GO:0007584 | response to nutrient | 5 | 7.462687 | 0.000133 | NQO1, CNR1, PPARG, HMGCR, MTOR |
| GO-BP | GO:0043278 | response to morphine | 4 | 5.970149 | 0.00016 | CNR1, MDM2, DRD2, MTOR |
| GO-BP | GO:0032148 | activation of protein kinase B activity | 4 | 5.970149 | 0.00016 | SRC, IGF1, FGF1, MTOR |
| GO-BP | GO:0009410 | response to xenobiotic stimulus | 7 | 10.44776 | 0.000169 | ABCB1, SRC, CASP3, MMP2, HTR2A, DRD2, RHOA |
| GO-BP | GO:0060045 | positive regulation of cardiac muscle cell proliferation | 4 | 5.970149 | 0.000193 | ERBB4, MAPK1, FGF2, FGFR2 |
| GO-BP | GO:0043388 | positive regulation of DNA binding | 4 | 5.970149 | 0.000193 | PPARG, IGF1, JAK2, MMP9 |
| GO-BP | GO:0019221 | cytokine-mediated signaling pathway | 6 | 8.955224 | 0.000194 | STAT3, KIT, AKT1, PTPN11, JAK2, F3 |
| GO-BP | GO:0043542 | endothelial cell migration | 4 | 5.970149 | 0.000212 | PIK3CA, NOS3, RHOA, TGFBR1 |
| GO-BP | GO:0046427 | positive regulation of JAK-STAT cascade | 4 | 5.970149 | 0.000273 | ERBB4, KIT, F2, JAK2 |
| GO-BP | GO:0045907 | positive regulation of vasoconstriction | 4 | 5.970149 | 0.000295 | AKT1, HMGCR, HTR2A, EGFR |
| GO-BP | GO:0042220 | response to cocaine | 4 | 5.970149 | 0.000319 | CNR1, MDM2, DRD2, MTOR |
| GO-BP | GO:0071276 | cellular response to cadmium ion | 4 | 5.970149 | 0.000371 | MAPK1, AKT1, MMP9, EGFR |
| GO-BP | GO:0031295 | T cell costimulation | 4 | 5.970149 | 0.000371 | PIK3CA, SRC, AKT1, PTPN11 |
| GO-BP | GO:0030574 | collagen catabolic process | 4 | 5.970149 | 0.000371 | MMP12, MMP2, MMP3, MMP9 |
| GO-BP | GO:0045822 | negative regulation of heart contraction | 3 | 4.477612 | 0.000408 | PDE4D, JAK2, IL2 |
| GO-BP | GO:0007281 | germ cell development | 4 | 5.970149 | 0.000427 | RARA, AKT1, MTOR, BCL2L1 |
| GO-BP | GO:0045747 | positive regulation of Notch signaling pathway | 4 | 5.970149 | 0.000489 | SRC, NOS3, STAT3, KIT |
| GO-BP | GO:0042177 | negative regulation of protein catabolic process | 4 | 5.970149 | 0.000489 | NQO1, NOS2, HMGCR, EGFR |
| GO-BP | GO:0014065 | phosphatidylinositol 3-kinase signaling | 4 | 5.970149 | 0.000489 | PIK3CA, AKT1, IGF1, HTR2A |
| GO-BP | GO:0045792 | negative regulation of cell size | 3 | 4.477612 | 0.000509 | AKT1, RHOA, MTOR |
| GO-BP | GO:0071222 | cellular response to lipopolysaccharide | 6 | 8.955224 | 0.000517 | NOS2, SRC, RARA, EPHB2, JAK2, RHOA |
| GO-BP | GO:0030334 | regulation of cell migration | 5 | 7.462687 | 0.000529 | ERBB4, AKT1, FGF1, FGF2, RHOA |
| GO-BP | GO:0001837 | epithelial to mesenchymal transition | 4 | 5.970149 | 0.000556 | IGF1, TGFBR1, FGFR2, FGFR1 |
| GO-BP | GO:0008584 | male gonad development | 5 | 7.462687 | 0.000586 | KIT, REN, ESR1, TGFBR1, BCL2L1 |
| GO-BP | GO:0071364 | cellular response to epidermal growth factor stimulus | 4 | 5.970149 | 0.000591 | ERBB4, AKT1, PTPN11, EGFR |
| GO-BP | GO:0071492 | cellular response to UV-A | 3 | 4.477612 | 0.00062 | MMP2, MMP3, MMP9 |
| GO-BP | GO:0070723 | response to cholesterol | 3 | 4.477612 | 0.00062 | F7, HMGCR, TGFBR1 |
| GO-BP | GO:0051781 | positive regulation of cell division | 4 | 5.970149 | 0.000628 | FGF1, FGF2, FGFR2, VEGFA |
| GO-BP | GO:0045429 | positive regulation of nitric oxide biosynthetic process | 4 | 5.970149 | 0.000628 | AKT1, JAK2, ESR1, MTOR |
| GO-BP | GO:0009755 | hormone-mediated signaling pathway | 4 | 5.970149 | 0.000707 | RARA, REN, PTPN11, PPARG |
| GO-BP | GO:0008542 | visual learning | 4 | 5.970149 | 0.000707 | KIT, HMGCR, DRD2, MTOR |
| GO-BP | GO:0051091 | positive regulation of sequence-specific DNA binding transcription factor activity | 5 | 7.462687 | 0.000736 | KIT, AKT1, PPARG, JAK2, ESR1 |
| GO-BP | GO:0043276 | anoikis | 3 | 4.477612 | 0.000743 | PIK3CA, AKT1, MTOR |
| GO-BP | GO:0009408 | response to heat | 4 | 5.970149 | 0.000791 | NOS3, AKT1, IGF1, MTOR |
| GO-BP | GO:0035556 | intracellular signal transduction | 8 | 11.9403 | 0.00086 | MAPK10, SRC, KIT, MAPK1, AKT1, JAK2, DRD2, TGFBR1 |
| GO-BP | GO:0051918 | negative regulation of fibrinolysis | 3 | 4.477612 | 0.000876 | PLAU, PLG, F2 |
| GO-BP | GO:0048011 | neurotrophin TRK receptor signaling pathway | 3 | 4.477612 | 0.000876 | SRC, CASP3, PTPN11 |
| GO-BP | GO:0070371 | ERK1 and ERK2 cascade | 4 | 5.970149 | 0.000881 | KDR, MAPK1, IGF1, FGF2 |
| GO-BP | GO:0008285 | negative regulation of cell proliferation | 8 | 11.9403 | 0.001014 | ERBB4, NOS3, STAT3, RARA, PLG, JAK2, DRD2, FGF2 |
| GO-BP | GO:0070102 | interleukin-6-mediated signaling pathway | 3 | 4.477612 | 0.00102 | SRC, STAT3, JAK2 |
| GO-BP | GO:0060644 | mammary gland epithelial cell differentiation | 3 | 4.477612 | 0.00102 | ERBB4, AKT1, FGF2 |
| GO-BP | GO:0016310 | phosphorylation | 5 | 7.462687 | 0.001024 | PIK3CA, STAT3, AKT1, EPHB2, MTOR |
| GO-BP | GO:0071230 | cellular response to amino acid stimulus | 4 | 5.970149 | 0.001082 | MMP2, EGFR, MTOR, BCL2L1 |
| GO-BP | GO:0016242 | negative regulation of macroautophagy | 3 | 4.477612 | 0.001174 | PIK3CA, AKT1, MTOR |
| GO-BP | GO:0043525 | positive regulation of neuron apoptotic process | 4 | 5.970149 | 0.001309 | BACE1, NQO1, CASP3, RHOA |
| GO-BP | GO:0038084 | vascular endothelial growth factor signaling pathway | 3 | 4.477612 | 0.001339 | PIK3CA, KDR, VEGFA |
| GO-BP | GO:0032757 | positive regulation of interleukin-8 production | 4 | 5.970149 | 0.001432 | NOS2, STAT3, F3, ELANE |
| GO-BP | GO:0006468 | protein phosphorylation | 8 | 11.9403 | 0.001577 | MAPK10, RARA, MAPK1, AKT1, JAK2, TGFBR1, MTOR, FGFR1 |
| GO-BP | GO:0000122 | negative regulation of transcription from RNA polymerase II promoter | 11 | 16.41791 | 0.001582 | MMP12, MAPK10, TRPV4, STAT3, MDM2, RARA, PPARG, ESR1, ELANE, FGFR2, VEGFA |
| GO-BP | GO:0060749 | mammary gland alveolus development | 3 | 4.477612 | 0.0017 | ERBB4, ESR1, VEGFA |
| GO-BP | GO:0043154 | negative regulation of cysteine-type endopeptidase activity involved in apoptotic process | 4 | 5.970149 | 0.001701 | SRC, MDM2, AKT1, VEGFA |
| GO-BP | GO:0018107 | peptidyl-threonine phosphorylation | 4 | 5.970149 | 0.001847 | MAPK1, AKT1, TGFBR1, MTOR |
| GO-BP | GO:2000811 | negative regulation of anoikis | 3 | 4.477612 | 0.001895 | PIK3CA, SRC, BCL2L1 |
| GO-BP | GO:2000573 | positive regulation of DNA biosynthetic process | 3 | 4.477612 | 0.001895 | SRC, FGF2, VEGFA |
| GO-BP | GO:0071391 | cellular response to estrogen stimulus | 3 | 4.477612 | 0.001895 | MDM2, RARA, ESR1 |
| GO-BP | GO:0060391 | positive regulation of SMAD protein import into nucleus | 3 | 4.477612 | 0.001895 | PPARG, JAK2, TGFBR1 |
| GO-BP | GO:0042730 | fibrinolysis | 3 | 4.477612 | 0.001895 | PLAU, PLG, F2 |
| GO-BP | GO:0007413 | axonal fasciculation | 3 | 4.477612 | 0.001895 | CNR1, CASP3, EPHB2 |
| GO-BP | GO:0008360 | regulation of cell shape | 5 | 7.462687 | 0.001957 | KIT, KDR, F2, RHOA, VEGFA |
| GO-BP | GO:0000187 | activation of MAPK activity | 4 | 5.970149 | 0.002 | KIT, IGF1, FGF1, FGF2 |
| GO-BP | GO:0070301 | cellular response to hydrogen peroxide | 4 | 5.970149 | 0.00208 | NQO1, SRC, MDM2, PTPN11 |
| GO-BP | GO:0043524 | negative regulation of neuron apoptotic process | 5 | 7.462687 | 0.002098 | PIK3CA, KDR, JAK2, RHOA, BCL2L1 |
| GO-BP | GO:0060716 | labyrinthine layer blood vessel development | 3 | 4.477612 | 0.002101 | MAPK1, AKT1, PLG |
| GO-BP | GO:0007204 | positive regulation of cytosolic calcium ion concentration | 5 | 7.462687 | 0.002147 | TRPV4, HTR2A, JAK2, ESR1, IL2 |
| GO-BP | GO:0030154 | cell differentiation | 9 | 13.43284 | 0.002154 | SRC, RARA, AKT1, PPARG, JAK2, FGF1, FGF2, EGFR, VEGFA |
| GO-BP | GO:0016477 | cell migration | 6 | 8.955224 | 0.002189 | PIK3CA, ERBB4, KDR, MET, RHOA, FGFR1 |
| GO-BP | GO:0001659 | temperature homeostasis | 3 | 4.477612 | 0.002317 | STAT3, HTR2A, DRD2 |
| GO-BP | GO:0090201 | negative regulation of release of cytochrome c from mitochondria | 3 | 4.477612 | 0.002544 | AKT1, IGF1, BCL2L1 |
| GO-BP | GO:0038083 | peptidyl-tyrosine autophosphorylation | 3 | 4.477612 | 0.002544 | SRC, KDR, EGFR |
| GO-BP | GO:0031641 | regulation of myelination | 3 | 4.477612 | 0.002544 | RARA, AKT1, MTOR |
| GO-BP | GO:0001933 | negative regulation of protein phosphorylation | 4 | 5.970149 | 0.002691 | EPHB2, DRD2, IL2, MTOR |
| GO-BP | GO:0016485 | protein processing | 4 | 5.970149 | 0.002787 | BACE1, F7, CASP3, F3 |
| GO-BP | GO:0007399 | nervous system development | 7 | 10.44776 | 0.002914 | ERBB4, STAT3, EPHB2, FGF2, MET, TGFBR1, VEGFA |
| GO-BP | GO:0045821 | positive regulation of glycolytic process | 3 | 4.477612 | 0.003026 | IGF1, HTR2A, MTOR |
| GO-BP | GO:0008203 | cholesterol metabolic process | 2 | 5.970149 | 0.003085 | CYP2C9, GBA |
| GO-BP | GO:0060135 | maternal process involved in female pregnancy | 3 | 4.477612 | 0.003282 | CNR1, CBS, MTOR |
| GO-BP | GO:0045445 | myoblast differentiation | 3 | 4.477612 | 0.003282 | PLG, HMGCR, IGF1 |
| GO-BP | GO:0040014 | regulation of multicellular organism growth | 3 | 4.477612 | 0.003282 | PIK3CA, STAT3, IGF1 |
| GO-BP | GO:0071260 | cellular response to mechanical stimulus | 4 | 5.970149 | 0.003295 | AKT1, PTPN11, FGF2, EGFR |
| GO-BP | GO:0009636 | response to toxic substance | 4 | 5.970149 | 0.003295 | NQO1, MDM2, MAPK1, DRD2 |
| GO-BP | GO:0018105 | peptidyl-serine phosphorylation | 5 | 7.462687 | 0.003425 | SRC, MAPK1, AKT1, TGFBR1, MTOR |
| GO-BP | GO:0001935 | endothelial cell proliferation | 3 | 4.477612 | 0.003547 | TEK, FGF2, TGFBR1 |
| GO-BP | GO:0033138 | positive regulation of peptidyl-serine phosphorylation | 4 | 5.970149 | 0.00374 | PIK3CA, AKT1, EGFR, VEGFA |
| GO-BP | GO:0008283 | cell proliferation | 5 | 7.462687 | 0.003774 | SRC, STAT3, AKT1, IGF1, BCL2L1 |
| GO-BP | GO:1905564 | positive regulation of vascular endothelial cell proliferation | 3 | 4.477612 | 0.003823 | STAT3, FGF2, FGFR1 |
| GO-BP | GO:0002052 | positive regulation of neuroblast proliferation | 3 | 4.477612 | 0.004108 | DRD2, FGF2, VEGFA |
| GO-BP | GO:1903672 | positive regulation of sprouting angiogenesis | 3 | 4.477612 | 0.004402 | FGF1, FGF2, VEGFA |
| GO-BP | GO:0006915 | apoptotic process | 8 | 11.9403 | 0.004587 | CASP3, MDM2, MAPK1, JAK2, MMP9, TGFBR1, FGFR2, BCL2L1 |
| GO-BP | GO:0051602 | response to electrical stimulus | 3 | 4.477612 | 0.004706 | NQO1, SRC, MMP2 |
| GO-BP | GO:0048010 | vascular endothelial growth factor receptor signaling pathway | 3 | 4.477612 | 0.004706 | SRC, KDR, VEGFA |
| GO-BP | GO:0043200 | response to amino acid | 3 | 4.477612 | 0.00502 | CASP3, RHOA, MTOR |
| GO-BP | GO:0032755 | positive regulation of interleukin-6 production | 4 | 5.970149 | 0.005289 | NOS2, TRPV4, STAT3, PTPN11 |
| GO-BP | GO:0046677 | response to antibiotic | 3 | 4.477612 | 0.005343 | CASP3, MDM2, JAK2 |
| GO-BP | GO:1900182 | positive regulation of protein localization to nucleus | 3 | 4.477612 | 0.005675 | SRC, AKT1, F2 |
| GO-BP | GO:0048705 | skeletal system morphogenesis | 3 | 4.477612 | 0.005675 | TGFBR1, FGFR2, FGFR1 |
| GO-BP | GO:0042476 | odontogenesis | 3 | 4.477612 | 0.006016 | SRC, RHOA, FGFR2 |
| GO-BP | GO:0035924 | cellular response to vascular endothelial growth factor stimulus | 3 | 4.477612 | 0.006016 | KDR, AKT1, VEGFA |
| GO-BP | GO:0032760 | positive regulation of tumor necrosis factor production | 4 | 5.970149 | 0.006032 | STAT3, PTPN11, EPHB2, JAK2 |
| GO-BP | GO:0001701 | in utero embryonic development | 5 | 7.462687 | 0.006086 | NOS3, TGFBR1, FGFR2, BCL2L1, VEGFA |
| GO-BP | GO:0090263 | positive regulation of canonical Wnt signaling pathway | 4 | 5.970149 | 0.006187 | SRC, FGF2, EGFR, FGFR2 |
| GO-BP | GO:0050729 | positive regulation of inflammatory response | 4 | 5.970149 | 0.006346 | TRPV4, JAK2, EGFR, IL2 |
| GO-BP | GO:0006874 | cellular calcium ion homeostasis | 4 | 5.970149 | 0.006346 | TRPV4, HTR2A, DRD2, ELANE |
| GO-BP | GO:0090050 | positive regulation of cell migration involved in sprouting angiogenesis | 3 | 4.477612 | 0.006367 | KDR, FGF2, VEGFA |
| GO-BP | GO:0002027 | regulation of heart rate | 3 | 4.477612 | 0.006367 | PDE4D, MDM2, DRD2 |
| GO-BP | GO:0048469 | cell maturation | 3 | 4.477612 | 0.006727 | REN, PPARG, VEGFA |
| GO-BP | GO:1902728 | positive regulation of growth factor dependent skeletal muscle satellite cell proliferation | 2 | 2.985075 | 0.006825 | STAT3, JAK2 |
| GO-BP | GO:2001237 | negative regulation of extrinsic apoptotic signaling pathway | 3 | 4.477612 | 0.007096 | SRC, IGF1, TGFBR1 |
| GO-BP | GO:0001974 | blood vessel remodeling | 3 | 4.477612 | 0.007473 | CBS, NOS3, MDM2 |
| GO-BP | GO:0046326 | positive regulation of glucose import | 3 | 4.477612 | 0.00786 | AKT1, PTPN11, IGF1 |
| GO-BP | GO:0031663 | lipopolysaccharide-mediated signaling pathway | 3 | 4.477612 | 0.00786 | NOS3, MAPK1, AKT1 |
| GO-BP | GO:0001822 | kidney development | 4 | 5.970149 | 0.00806 | REN, RHOA, TGFBR1, VEGFA |
| GO-BP | GO:0048286 | lung alveolus development | 3 | 4.477612 | 0.008256 | MMP12, KDR, FGFR2 |
| GO-BP | GO:0071392 | cellular response to estradiol stimulus | 3 | 4.477612 | 0.009073 | MMP2, ESR1, EGFR |
| GO-BP | GO:0006953 | acute-phase response | 3 | 4.477612 | 0.009073 | SERPINA1, STAT3, F2 |
| GO-BP | GO:0003007 | heart morphogenesis | 3 | 4.477612 | 0.009073 | EPHB4, MTOR, VEGFA |
| GO-BP | GO:0042981 | regulation of apoptotic process | 5 | 7.462687 | 0.009192 | RARA, AKT1, JAK2, ESR1, BCL2L1 |
| GO-BP | GO:0097009 | energy homeostasis | 3 | 4.477612 | 0.009495 | PIK3CA, TRPV4, STAT3 |
| GO-BP | GO:0051209 | release of sequestered calcium ion into cytosol | 3 | 4.477612 | 0.009495 | HTR2A, DRD2, FGF2 |
| GO-BP | GO:0050728 | negative regulation of inflammatory response | 4 | 5.970149 | 0.009816 | PPARG, TEK, IL2, ELANE |
| GO-BP | GO:0050918 | positive chemotaxis | 3 | 4.477612 | 0.009925 | FGF2, MET, VEGFA |
| GO-BP | GO:0045454 | cell redox homeostasis | 3 | 4.477612 | 0.009925 | NQO1, NOS2, NOS3 |
| GO-BP | GO:2000544 | regulation of endothelial cell chemotaxis to fibroblast growth factor | 2 | 2.985075 | 0.01022 | FGF1, FGF2 |
| GO-BP | GO:0070141 | response to UV-A | 2 | 2.985075 | 0.01022 | AKT1, EGFR |
| GO-BP | GO:0060523 | prostate epithelial cord elongation | 2 | 2.985075 | 0.01022 | ESR1, FGFR2 |
| GO-BP | GO:0047484 | regulation of response to osmotic stress | 2 | 2.985075 | 0.01022 | ABCB1, TRPV4 |
| GO-BP | GO:0033688 | regulation of osteoblast proliferation | 2 | 2.985075 | 0.01022 | RHOA, FGFR2 |
| GO-BP | GO:0021769 | orbitofrontal cortex development | 2 | 2.985075 | 0.01022 | DRD2, FGFR2 |
| GO-BP | GO:0010641 | positive regulation of platelet-derived growth factor receptor signaling pathway | 2 | 2.985075 | 0.01022 | F7, F3 |
| GO-BP | GO:0006743 | ubiquinone metabolic process | 2 | 2.985075 | 0.01022 | NQO1, HMGCR |
| GO-BP | GO:0042789 | mRNA transcription from RNA polymerase II promoter | 3 | 4.477612 | 0.010364 | STAT3, RARA, PPARG |
| GO-BP | GO:0009725 | response to hormone | 3 | 4.477612 | 0.010364 | NQO1, NOS2, NOS3 |
| GO-BP | GO:1904646 | cellular response to beta-amyloid | 3 | 4.477612 | 0.011267 | BACE1, IGF1, EPHB2 |
| GO-BP | GO:0001501 | skeletal system development | 4 | 5.970149 | 0.011323 | IGF1, MMP9, TGFBR1, FGFR1 |
| GO-BP | GO:1902895 | positive regulation of pri-miRNA transcription from RNA polymerase II promoter | 3 | 4.477612 | 0.011732 | STAT3, PPARG, FGF2 |
| GO-BP | GO:0048015 | phosphatidylinositol-mediated signaling | 3 | 4.477612 | 0.011732 | PIK3CA, IGF1, FGFR1 |
| GO-BP | GO:0030218 | erythrocyte differentiation | 3 | 4.477612 | 0.013174 | CASP3, KIT, JAK2 |
| GO-BP | GO:2000546 | positive regulation of endothelial cell chemotaxis to fibroblast growth factor | 2 | 2.985075 | 0.013604 | FGF2, FGFR1 |
| GO-BP | GO:1901532 | regulation of hematopoietic progenitor cell differentiation | 2 | 2.985075 | 0.013604 | RARA, KDR |
| GO-BP | GO:0070945 | neutrophil mediated killing of gram-negative bacterium | 2 | 2.985075 | 0.013604 | F2, ELANE |
| GO-BP | GO:0060978 | angiogenesis involved in coronary vascular morphogenesis | 2 | 2.985075 | 0.013604 | FGF2, TGFBR1 |
| GO-BP | GO:0051919 | positive regulation of fibrinolysis | 2 | 2.985075 | 0.013604 | F11, PLG |
| GO-BP | GO:0038127 | ERBB signaling pathway | 2 | 2.985075 | 0.013604 | MAPK1, PTPN11 |
| GO-BP | GO:0010749 | regulation of nitric oxide mediated signal transduction | 2 | 2.985075 | 0.013604 | CBS, VEGFA |
| GO-BP | GO:0072089 | stem cell proliferation | 3 | 4.477612 | 0.013671 | ABCB1, KDR, FGF2 |
| GO-BP | GO:0045737 | positive regulation of cyclin-dependent protein serine/threonine kinase activity | 3 | 4.477612 | 0.013671 | SRC, AKT1, EGFR |
| GO-BP | GO:2000300 | regulation of synaptic vesicle exocytosis | 3 | 4.477612 | 0.014176 | BACE1, HTR2A, DRD2 |
| GO-BP | GO:0051496 | positive regulation of stress fiber assembly | 3 | 4.477612 | 0.01521 | RHOA, TGFBR1, MTOR |
| GO-BP | GO:0045600 | positive regulation of fat cell differentiation | 3 | 4.477612 | 0.015739 | AKT1, PPARG, HTR2A |
| GO-BP | GO:1903078 | positive regulation of protein localization to plasma membrane | 3 | 4.477612 | 0.016276 | AKT1, EPHB2, EGFR |
| GO-BP | GO:0048146 | positive regulation of fibroblast proliferation | 3 | 4.477612 | 0.016276 | IGF1, ESR1, EGFR |
| GO-BP | GO:0034446 | substrate adhesion-dependent cell spreading | 3 | 4.477612 | 0.01682 | SRC, TEK, RHOA |
| GO-BP | GO:0014823 | response to activity | 3 | 4.477612 | 0.01682 | PIK3CA, MMP2, MTOR |
| GO-BP | GO:1905278 | positive regulation of epithelial tube formation | 2 | 2.985075 | 0.016977 | FGF2, VEGFA |
| GO-BP | GO:0060591 | chondroblast differentiation | 2 | 2.985075 | 0.016977 | RARA, FGF2 |
| GO-BP | GO:0038033 | positive regulation of endothelial cell chemotaxis by VEGF-activated vascular endothelial growth factor receptor signaling pathway | 2 | 2.985075 | 0.016977 | KDR, VEGFA |
| GO-BP | GO:0036324 | vascular endothelial growth factor receptor-2 signaling pathway | 2 | 2.985075 | 0.016977 | KDR, VEGFA |
| GO-BP | GO:0033602 | negative regulation of dopamine secretion | 2 | 2.985075 | 0.016977 | CNR1, DRD2 |
| GO-BP | GO:0031999 | negative regulation of fatty acid beta-oxidation | 2 | 2.985075 | 0.016977 | CNR1, AKT1 |
| GO-BP | GO:0031532 | actin cytoskeleton reorganization | 3 | 4.477612 | 0.017372 | TRPV4, KIT, RHOA |
| GO-BP | GO:0042127 | regulation of cell proliferation | 4 | 5.970149 | 0.018634 | NOS2, PLAU, STAT3, KIT |
| GO-BP | GO:0030198 | extracellular matrix organization | 4 | 5.970149 | 0.018934 | MMP12, MMP2, MMP3, MMP9 |
| GO-BP | GO:0030168 | platelet activation | 3 | 4.477612 | 0.020247 | PIK3CA, SRC, F2 |
| GO-BP | GO:0060527 | prostate epithelial cord arborization involved in prostate glandular acinus morphogenesis | 2 | 2.985075 | 0.020338 | ESR1, FGFR2 |
| GO-BP | GO:0048755 | branching morphogenesis of a nerve | 2 | 2.985075 | 0.020338 | DRD2, FGFR2 |
| GO-BP | GO:0048170 | positive regulation of long-term neuronal synaptic plasticity | 2 | 2.985075 | 0.020338 | KIT, EPHB2 |
| GO-BP | GO:0003100 | regulation of systemic arterial blood pressure by endothelin | 2 | 2.985075 | 0.020338 | NOS3, RHOA |
| GO-BP | GO:0042752 | regulation of circadian rhythm | 3 | 4.477612 | 0.021449 | MAPK10, PPARG, MTOR |
| GO-BP | GO:0035690 | cellular response to drug | 3 | 4.477612 | 0.022061 | NOS2, REN, EGFR |
| GO-BP | GO:0043457 | regulation of cellular respiration | 2 | 2.985075 | 0.023688 | PIK3CA, NOS2 |
| GO-BP | GO:0006527 | arginine catabolic process | 2 | 2.985075 | 0.023688 | NOS2, NOS3 |
| GO-BP | GO:0007623 | circadian rhythm | 3 | 4.477612 | 0.023939 | F7, NOS2, EGFR |
| GO-BP | GO:0006909 | phagocytosis | 3 | 4.477612 | 0.023939 | PIK3CA, MET, ELANE |
| GO-BP | GO:0010977 | negative regulation of neuron projection development | 3 | 4.477612 | 0.02458 | TRPV4, MDM2, RHOA |
| GO-BP | GO:0009791 | post-embryonic development | 3 | 4.477612 | 0.02458 | TGFBR1, FGFR2, MTOR |
| GO-BP | GO:0071300 | cellular response to retinoic acid | 3 | 4.477612 | 0.026543 | RARA, PPARG, FGFR2 |
| GO-BP | GO:0071312 | cellular response to alkaloid | 2 | 2.985075 | 0.027026 | MDM2, BCL2L1 |
| GO-BP | GO:0061042 | vascular wound healing | 2 | 2.985075 | 0.027026 | KDR, VEGFA |
| GO-BP | GO:0051093 | negative regulation of developmental process | 2 | 2.985075 | 0.027026 | KIT, BCL2L1 |
| GO-BP | GO:0048762 | mesenchymal cell differentiation | 2 | 2.985075 | 0.027026 | TGFBR1, FGFR2 |
| GO-BP | GO:0010544 | negative regulation of platelet activation | 2 | 2.985075 | 0.027026 | NOS3, F2 |
| GO-BP | GO:0010518 | positive regulation of phospholipase activity | 2 | 2.985075 | 0.027026 | FGFR2, FGFR1 |
| GO-BP | GO:0048511 | rhythmic process | 3 | 4.477612 | 0.028567 | MAPK10, PPARG, MTOR |
| GO-BP | GO:0032147 | activation of protein kinase activity | 3 | 4.477612 | 0.029255 | PIK3CA, DRD2, VEGFA |
| GO-BP | GO:0032729 | positive regulation of interferon-gamma production | 3 | 4.477612 | 0.02995 | PDE4D, JAK2, IL2 |
| GO-BP | GO:2000641 | regulation of early endosome to late endosome transport | 2 | 2.985075 | 0.030353 | SRC, MAPK1 |
| GO-BP | GO:2000242 | negative regulation of reproductive process | 2 | 2.985075 | 0.030353 | KIT, BCL2L1 |
| GO-BP | GO:0060982 | coronary artery morphogenesis | 2 | 2.985075 | 0.030353 | TGFBR1, VEGFA |
| GO-BP | GO:0060351 | cartilage development involved in endochondral bone morphogenesis | 2 | 2.985075 | 0.030353 | CBS, TRPV4 |
| GO-BP | GO:0048771 | tissue remodeling | 2 | 2.985075 | 0.030353 | MMP2, PLG |
| GO-BP | GO:0042756 | drinking behavior | 2 | 2.985075 | 0.030353 | REN, DRD2 |
| GO-BP | GO:0034405 | response to fluid shear stress | 2 | 2.985075 | 0.030353 | NOS3, AKT1 |
| GO-BP | GO:0021795 | cerebral cortex cell migration | 2 | 2.985075 | 0.030353 | RHOA, EGFR |
| GO-BP | GO:0008354 | germ cell migration | 2 | 2.985075 | 0.030353 | KIT, TGFBR1 |
| GO-BP | GO:0001542 | ovulation from ovarian follicle | 2 | 2.985075 | 0.030353 | NOS3, MMP2 |
| GO-BP | GO:0008217 | regulation of blood pressure | 3 | 4.477612 | 0.030651 | NOS3, REN, PPARG |
| GO-BP | GO:0006919 | activation of cysteine-type endopeptidase activity involved in apoptotic process | 3 | 4.477612 | 0.032073 | PPARG, JAK2, F3 |
| GO-BP | GO:0045727 | positive regulation of translation | 3 | 4.477612 | 0.033521 | MAPK1, RHOA, MTOR |
| GO-BP | GO:2001028 | positive regulation of endothelial cell chemotaxis | 2 | 2.985075 | 0.033669 | FGF2, MET |
| GO-BP | GO:2000048 | negative regulation of cell-cell adhesion mediated by cadherin | 2 | 2.985075 | 0.033669 | PLG, VEGFA |
| GO-BP | GO:0060397 | JAK-STAT cascade involved in growth hormone signaling pathway | 2 | 2.985075 | 0.033669 | STAT3, JAK2 |
| GO-BP | GO:0060259 | regulation of feeding behavior | 2 | 2.985075 | 0.033669 | CNR1, STAT3 |
| GO-BP | GO:0051272 | positive regulation of cellular component movement | 2 | 2.985075 | 0.033669 | TGFBR1, VEGFA |
| GO-BP | GO:0043586 | tongue development | 2 | 2.985075 | 0.033669 | KIT, EGFR |
| GO-BP | GO:0043267 | negative regulation of potassium ion transport | 2 | 2.985075 | 0.033669 | NOS3, HTR2A |
| GO-BP | GO:0010907 | positive regulation of glucose metabolic process | 2 | 2.985075 | 0.033669 | SRC, AKT1 |
| GO-BP | GO:1903715 | regulation of aerobic respiration | 2 | 2.985075 | 0.036974 | TRPV4, AKT1 |
| GO-BP | GO:1902533 | positive regulation of intracellular signal transduction | 2 | 2.985075 | 0.036974 | TEK, FGF1 |
| GO-BP | GO:0099527 | postsynapse to nucleus signaling pathway | 2 | 2.985075 | 0.036974 | STAT3, JAK2 |
| GO-BP | GO:0071803 | positive regulation of podosome assembly | 2 | 2.985075 | 0.036974 | SRC, RHOA |
| GO-BP | GO:0071679 | commissural neuron axon guidance | 2 | 2.985075 | 0.036974 | EPHB2, VEGFA |
| GO-BP | GO:0060020 | Bergmann glial cell differentiation | 2 | 2.985075 | 0.036974 | MAPK1, PTPN11 |
| GO-BP | GO:0051967 | negative regulation of synaptic transmission, glutamatergic | 2 | 2.985075 | 0.036974 | HTR2A, DRD2 |
| GO-BP | GO:0032754 | positive regulation of interleukin-5 production | 2 | 2.985075 | 0.036974 | PDE4D, RARA |
| GO-BP | GO:0031639 | plasminogen activation | 2 | 2.985075 | 0.036974 | PLAU, F11 |
| GO-BP | GO:0030193 | regulation of blood coagulation | 2 | 2.985075 | 0.036974 | EPHB2, F2 |
| GO-BP | GO:0001759 | organ induction | 2 | 2.985075 | 0.036974 | FGF1, FGF2 |
| GO-BP | GO:0007409 | axonogenesis | 3 | 4.477612 | 0.03725 | PTPN11, DRD2, FGFR2 |
| GO-BP | GO:0035264 | multicellular organism growth | 3 | 4.477612 | 0.038014 | RARA, PTPN11, MTOR |
| GO-BP | GO:0001889 | liver development | 3 | 4.477612 | 0.039561 | PIK3CA, RARA, MET |
| GO-BP | GO:0097011 | cellular response to granulocyte macrophage colony-stimulating factor stimulus | 2 | 2.985075 | 0.040268 | MAPK1, AKT1 |
| GO-BP | GO:0071470 | cellular response to osmotic stress | 2 | 2.985075 | 0.040268 | TRPV4, MTOR |
| GO-BP | GO:0061051 | positive regulation of cell growth involved in cardiac muscle cell development | 2 | 2.985075 | 0.040268 | IGF1, MTOR |
| GO-BP | GO:0051549 | positive regulation of keratinocyte migration | 2 | 2.985075 | 0.040268 | MMP9, MTOR |
| GO-BP | GO:0046425 | regulation of JAK-STAT cascade | 2 | 2.985075 | 0.040268 | EPHB2, JAK2 |
| GO-BP | GO:0043117 | positive regulation of vascular permeability | 2 | 2.985075 | 0.040268 | TRPV4, VEGFA |
| GO-BP | GO:0031284 | positive regulation of guanylate cyclase activity | 2 | 2.985075 | 0.040268 | NOS2, NOS3 |
| GO-BP | GO:0005979 | regulation of glycogen biosynthetic process | 2 | 2.985075 | 0.040268 | AKT1, MTOR |
| GO-BP | GO:0030855 | epithelial cell differentiation | 3 | 4.477612 | 0.041131 | PPARG, FGFR2, VEGFA |
| GO-BP | GO:0045666 | positive regulation of neuron differentiation | 3 | 4.477612 | 0.041924 | RARA, RHOA, FGFR1 |
| GO-BP | GO:2001223 | negative regulation of neuron migration | 2 | 2.985075 | 0.043551 | STAT3, DRD2 |
| GO-BP | GO:0060396 | growth hormone receptor signaling pathway | 2 | 2.985075 | 0.043551 | STAT3, JAK2 |
| GO-BP | GO:0060134 | prepulse inhibition | 2 | 2.985075 | 0.043551 | BACE1, DRD2 |
| GO-BP | GO:0051974 | negative regulation of telomerase activity | 2 | 2.985075 | 0.043551 | SRC, PPARG |
| GO-BP | GO:0051450 | myoblast proliferation | 2 | 2.985075 | 0.043551 | SRC, IGF1 |
| GO-BP | GO:0050435 | beta-amyloid metabolic process | 2 | 2.985075 | 0.043551 | BACE1, REN |
| GO-BP | GO:0046902 | regulation of mitochondrial membrane permeability | 2 | 2.985075 | 0.043551 | STAT3, BCL2L1 |
| GO-BP | GO:0043114 | regulation of vascular permeability | 2 | 2.985075 | 0.043551 | SRC, TEK |
| GO-BP | GO:0033628 | regulation of cell adhesion mediated by integrin | 2 | 2.985075 | 0.043551 | PLAU, PTPN11 |
| GO-BP | GO:0019430 | removal of superoxide radicals | 2 | 2.985075 | 0.043551 | NQO1, NOS3 |
| GO-BP | GO:0007179 | transforming growth factor beta receptor signaling pathway | 3 | 4.477612 | 0.046807 | SRC, STAT3, TGFBR1 |
| GO-BP | GO:0060416 | response to growth hormone | 2 | 2.985075 | 0.046822 | F7, AKT1 |
| GO-BP | GO:0007260 | tyrosine phosphorylation of STAT protein | 2 | 2.985075 | 0.046822 | JAK2, IL2 |
| GO-BP | GO:0006805 | xenobiotic metabolic process | 3 | 4.477612 | 0.04764 | NQO1, CYP2C9, ABCB1 |
| GO-BP | GO:0006606 | protein import into nucleus | 3 | 4.477612 | 0.048478 | MMP12, STAT3, AKT1 |
| GO-CC | GO:0005576 | extracellular region | 31 | 46.26866 | 3.08E-13 | SERPINA1, C1R, PLG, FGF1, FGF2, ABO, TTR, PLAU, ERBB4, KDR, MAPK1, EPHB2, EPHB4, ELANE, F10, MMP2, MMP3, F11, IGF1, F2, MMP9, IL2, VEGFA, MMP12, F7, ALB, REN, TEK, MET, FGFR2, FGFR1 |
| GO-CC | GO:0043235 | receptor complex | 12 | 17.91045 | 9.06E-11 | ERBB4, KIT, KDR, PPARG, TEK, EPHB2, MET, EPHB4, EGFR, TGFBR1, FGFR2, FGFR1 |
| GO-CC | GO:0005886 | plasma membrane | 42 | 62.68657 | 1.2E-10 | ABCB1, SRC, WAS, PLG, HTR2A, EGFR, PLAU, ERBB4, CNR1, KDR, AKT1, MAPK1, EPHB2, JAK2, DRD2, EPHB4, NOS2, F10, NOS3, PDE4D, MMP2, F11, STAT3, F2, F3, ESR1, RHOA, TGFBR1, BACE1, MAPK10, CYP2C9, F7, PIK3CA, TRPV4, KIT, RARA, MDM2, REN, TEK, MET, FGFR2, FGFR1 |
| GO-CC | GO:0009986 | cell surface | 15 | 22.38806 | 1.19E-08 | ABCB1, PLG, F3, EGFR, TGFBR1, VEGFA, BACE1, PLAU, TRPV4, RARA, TEK, EPHB2, MET, ELANE, FGFR2 |
| GO-CC | GO:0005615 | extracellular space | 24 | 35.8209 | 1.44E-08 | SERPINA1, F10, C1R, MMP2, F11, MMP3, PLG, IGF1, F2, FGF1, FGF2, F3, MMP9, EGFR, IL2, VEGFA, MMP12, F7, TTR, PLAU, KIT, ALB, REN, ELANE |
| GO-CC | GO:0098978 | glutamatergic synapse | 12 | 17.91045 | 2.66E-08 | MAPK10, ERBB4, CNR1, SRC, STAT3, PLG, HTR2A, EPHB2, JAK2, DRD2, RHOA, MTOR |
| GO-CC | GO:0045121 | membrane raft | 9 | 13.43284 | 1.42E-06 | BACE1, CNR1, SRC, CASP3, KDR, TEK, JAK2, EGFR, TGFBR1 |
| GO-CC | GO:0099056 | integral component of presynaptic membrane | 5 | 7.462687 | 6.01E-05 | ERBB4, CNR1, HTR2A, EPHB2, DRD2 |
| GO-CC | GO:0031093 | platelet alpha granule lumen | 5 | 7.462687 | 6.37E-05 | SERPINA1, ALB, PLG, IGF1, VEGFA |
| GO-CC | GO:0005887 | integral component of plasma membrane | 16 | 23.8806 | 6.75E-05 | HTR2A, F3, EGFR, BACE1, ERBB4, CNR1, TRPV4, KIT, KDR, TEK, EPHB2, DRD2, MET, EPHB4, FGFR2, FGFR1 |
| GO-CC | GO:0005901 | caveola | 5 | 7.462687 | 0.00011 | SRC, NOS3, MAPK1, HTR2A, JAK2 |
| GO-CC | GO:0005925 | focal adhesion | 8 | 11.9403 | 0.000418 | PLAU, SRC, TRPV4, MAPK1, TEK, JAK2, RHOA, EGFR |
| GO-CC | GO:0005788 | endoplasmic reticulum lumen | 7 | 10.44776 | 0.000444 | BACE1, F7, SERPINA1, F10, ALB, MAPK1, F2 |
| GO-CC | GO:0009925 | basal plasma membrane | 4 | 5.970149 | 0.000589 | ERBB4, TEK, MET, EGFR |
| GO-CC | GO:0005737 | cytoplasm | 31 | 46.26866 | 0.001046 | SRC, FGF1, FGF2, EGFR, CBS, CNR1, CASP3, AKT1, MAPK1, JAK2, ELANE, NQO1, NOS2, NOS3, STAT3, PTPN11, ESR1, MTOR, VEGFA, MMP12, MAPK10, CYP2C9, PIK3CA, ALB, RARA, MDM2, REN, PPARG, TEK, FGFR2, BCL2L1 |
| GO-CC | GO:0032991 | macromolecular complex | 9 | 13.43284 | 0.001792 | TTR, ALB, MDM2, RARA, MAPK1, AKT1, PTPN11, ESR1, EGFR |
| GO-CC | GO:0043025 | neuronal cell body | 7 | 10.44776 | 0.001959 | BACE1, NQO1, SRC, CASP3, HTR2A, EPHB2, MTOR |
| GO-CC | GO:0030425 | dendrite | 7 | 10.44776 | 0.003551 | BACE1, NQO1, RARA, HTR2A, EPHB2, DRD2, MTOR |
| GO-CC | GO:0032587 | ruffle membrane | 4 | 5.970149 | 0.003977 | SRC, TRPV4, RHOA, EGFR |
| GO-CC | GO:0012506 | vesicle membrane | 3 | 4.477612 | 0.004165 | NOS2, NOS3, WAS |
| GO-CC | GO:0005829 | cytosol | 29 | 43.28358 | 0.004288 | SRC, WAS, HTR2A, FGF1, ERBB4, CBS, CASP3, AKT1, MAPK1, EPHB2, JAK2, EPHB4, ELANE, NQO1, NOS2, NOS3, PDE4D, STAT3, PTPN11, ESR1, RHOA, MTOR, MAPK10, PIK3CA, RARA, MDM2, PPARG, FGFR1, BCL2L1 |
| GO-CC | GO:0030424 | axon | 6 | 8.955224 | 0.006041 | BACE1, MAPK1, HTR2A, EPHB2, DRD2, RHOA |
| GO-CC | GO:0098794 | postsynapse | 4 | 5.970149 | 0.006332 | AKT1, EPHB2, JAK2, RHOA |
| GO-CC | GO:1905286 | serine-type peptidase complex | 2 | 2.985075 | 0.006409 | F7, F3 |
| GO-CC | GO:0070062 | extracellular exosome | 15 | 22.38806 | 0.009731 | SERPINA1, ABCB1, C1R, SRC, F11, WAS, PLG, F2, MMP9, RHOA, TTR, PLAU, ALB, EPHB4, ELANE |
| GO-CC | GO:0031012 | extracellular matrix | 5 | 7.462687 | 0.009754 | MMP12, MMP2, MMP3, FGF1, VEGFA |
| GO-CC | GO:0005794 | Golgi apparatus | 10 | 14.92537 | 0.010361 | BACE1, SERPINA1, NOS3, ALB, KDR, MAPK1, EPHB2, ESR1, FGFR2, ABO |
| GO-CC | GO:0072562 | blood microparticle | 4 | 5.970149 | 0.011369 | C1R, ALB, PLG, F2 |
| GO-CC | GO:0099055 | integral component of postsynaptic membrane | 3 | 4.477612 | 0.011692 | HTR2A, EPHB2, DRD2 |
| GO-CC | GO:0031233 | intrinsic component of external side of plasma membrane | 2 | 2.985075 | 0.012778 | F10, F3 |
| GO-CC | GO:0005634 | nucleus | 29 | 43.28358 | 0.014204 | WAS, FGF1, FGF2, EGFR, ERBB4, CBS, CASP3, KDR, AKT1, MAPK1, JAK2, NOS2, NOS3, PDE4D, MMP2, STAT3, PTPN11, ESR1, RHOA, TGFBR1, MTOR, MMP12, MAPK10, ALB, RARA, MDM2, PPARG, FGFR2, FGFR1 |
| GO-CC | GO:0031982 | vesicle | 4 | 5.970149 | 0.018465 | F7, AKT1, RHOA, ABO |
| GO-CC | GO:0005768 | endosome | 5 | 7.462687 | 0.018912 | BACE1, KDR, RHOA, EGFR, TGFBR1 |
| GO-CC | GO:0031234 | extrinsic component of cytoplasmic side of plasma membrane | 3 | 4.477612 | 0.020174 | SRC, AKT1, RHOA |
| GO-CC | GO:0031410 | cytoplasmic vesicle | 5 | 7.462687 | 0.020513 | TRPV4, HTR2A, RHOA, FGFR2, FGFR1 |
| GO-CC | GO:0045335 | phagocytic vesicle | 3 | 4.477612 | 0.020734 | WAS, ELANE, MTOR |
| GO-CC | GO:0030027 | lamellipodium | 4 | 5.970149 | 0.023864 | PIK3CA, TRPV4, AKT1, RHOA |
| GO-CC | GO:0005911 | cell-cell junction | 4 | 5.970149 | 0.023864 | KIT, WAS, AKT1, TEK |
| GO-CC | GO:0098982 | GABA-ergic synapse | 3 | 4.477612 | 0.024226 | ERBB4, CNR1, DRD2 |
| GO-CC | GO:0016020 | membrane | 15 | 22.38806 | 0.029476 | ABCB1, PDE4D, F11, ESR1, EGFR, TGFBR1, MTOR, ABO, VEGFA, BACE1, PIK3CA, REN, JAK2, MET, FGFR2 |
| GO-CC | GO:0016324 | apical plasma membrane | 5 | 7.462687 | 0.032674 | ABCB1, PDE4D, TRPV4, TEK, EGFR |
| GO-CC | GO:0035578 | azurophil granule lumen | 3 | 4.477612 | 0.034603 | TTR, MAPK1, ELANE |
| GO-CC | GO:0048471 | perinuclear region of cytoplasm | 7 | 10.44776 | 0.035614 | PIK3CA, NOS2, SRC, PDE4D, RARA, PPARG, EGFR |
| GO-CC | GO:0005884 | actin filament | 3 | 4.477612 | 0.03671 | SRC, WAS, TEK |
| GO-CC | GO:0030054 | cell junction | 4 | 5.970149 | 0.038597 | SRC, KDR, RHOA, EGFR |
| GO-CC | GO:0005796 | Golgi lumen | 3 | 4.477612 | 0.045613 | F7, F10, F2 |
| GO-MF | GO:0042802 | identical protein binding | 32 | 47.76119 | 1.07E-15 | SERPINA1, C1R, WAS, HMGCR, HTR2A, FGF2, EGFR, TTR, CBS, CNR1, KDR, AKT1, MAPK1, EPHB2, JAK2, DRD2, NQO1, F11, STAT3, MMP9, ESR1, MTOR, VEGFA, TRPV4, ALB, MDM2, PPARG, TEK, MET, FGFR2, FGFR1, BCL2L1 |
| GO-MF | GO:0004714 | transmembrane receptor protein tyrosine kinase activity | 12 | 17.91045 | 2.36E-13 | ERBB4, SRC, KIT, KDR, TEK, EPHB2, JAK2, MET, EPHB4, EGFR, FGFR2, FGFR1 |
| GO-MF | GO:0004252 | serine-type endopeptidase activity | 13 | 19.40299 | 2.83E-12 | F10, C1R, MMP2, F11, MMP3, PLG, F2, F3, MMP9, MMP12, F7, PLAU, ELANE |
| GO-MF | GO:0004713 | protein tyrosine kinase activity | 11 | 16.41791 | 6.3E-12 | ERBB4, SRC, KIT, KDR, TEK, EPHB2, JAK2, MET, EGFR, FGFR2, FGFR1 |
| GO-MF | GO:0004175 | endopeptidase activity | 7 | 10.44776 | 5.87E-07 | MMP12, BACE1, MMP2, MMP3, PLG, MMP9, ELANE |
| GO-MF | GO:0005524 | ATP binding | 20 | 29.85075 | 8.28E-07 | ABCB1, SRC, EGFR, TGFBR1, MTOR, MAPK10, PIK3CA, ERBB4, TRPV4, KIT, KDR, AKT1, MAPK1, TEK, EPHB2, JAK2, MET, EPHB4, FGFR2, FGFR1 |
| GO-MF | GO:0019899 | enzyme binding | 11 | 16.41791 | 9.54E-07 | BACE1, SRC, CBS, PDE4D, MDM2, RARA, AKT1, PPARG, PLG, ESR1, EGFR |
| GO-MF | GO:0008201 | heparin binding | 8 | 11.9403 | 2.67E-06 | F11, F2, FGF1, FGF2, ELANE, FGFR2, FGFR1, VEGFA |
| GO-MF | GO:0005515 | protein binding | 61 | 91.04478 | 3.67E-06 | SERPINA1, HTR2A, FGF1, FGF2, PLAU, CASP3, KDR, AKT1, EPHB2, JAK2, EPHB4, PDE4D, MMP2, MMP3, F2, MMP9, F3, RHOA, TGFBR1, BACE1, F7, PIK3CA, KIT, RARA, PPARG, MET, ABCB1, SRC, C1R, WAS, PLG, HMGCR, EGFR, TTR, ERBB4, CBS, CNR1, MAPK1, DRD2, ELANE, NQO1, NOS2, F10, NOS3, F11, STAT3, PTPN11, IGF1, ESR1, MTOR, IL2, VEGFA, MAPK10, TRPV4, ALB, MDM2, REN, TEK, FGFR2, FGFR1, BCL2L1 |
| GO-MF | GO:0004672 | protein kinase activity | 10 | 14.92537 | 6.52E-06 | MAPK10, SRC, AKT1, TEK, JAK2, MET, EGFR, TGFBR1, FGFR2, MTOR |
| GO-MF | GO:0005102 | receptor binding | 10 | 14.92537 | 9.47E-06 | F7, SRC, STAT3, RARA, REN, PLG, EPHB2, F2, JAK2, TGFBR1 |
| GO-MF | GO:0019901 | protein kinase binding | 11 | 16.41791 | 1.05E-05 | TRPV4, STAT3, WAS, AKT1, PTPN11, JAK2, ESR1, RHOA, EGFR, MTOR, BCL2L1 |
| GO-MF | GO:0008233 | peptidase activity | 6 | 8.955224 | 2.88E-05 | BACE1, CASP3, MMP3, REN, MMP9, ELANE |
| GO-MF | GO:0020037 | heme binding | 6 | 8.955224 | 0.000185 | CYP2C9, NOS2, SRC, CBS, NOS3, JAK2 |
| GO-MF | GO:0005178 | integrin binding | 6 | 8.955224 | 0.00025 | SRC, KDR, IGF1, FGF1, FGF2, EGFR |
| GO-MF | GO:0008083 | growth factor activity | 6 | 8.955224 | 0.000288 | IGF1, F2, FGF1, FGF2, IL2, VEGFA |
| GO-MF | GO:0042169 | SH2 domain binding | 4 | 5.970149 | 0.000396 | SRC, TRPV4, KIT, JAK2 |
| GO-MF | GO:0030235 | nitric-oxide synthase regulator activity | 3 | 4.477612 | 0.000427 | AKT1, ESR1, EGFR |
| GO-MF | GO:0002020 | protease binding | 5 | 7.462687 | 0.000617 | SERPINA1, CASP3, KIT, F3, ELANE |
| GO-MF | GO:0042803 | protein homodimerization activity | 10 | 14.92537 | 0.000829 | NOS2, ERBB4, CBS, STAT3, KIT, AKT1, FGFR2, FGFR1, BCL2L1, VEGFA |
| GO-MF | GO:0004879 | RNA polymerase II transcription factor activity, ligand-activated sequence-specific DNA binding | 4 | 5.970149 | 0.000893 | STAT3, RARA, PPARG, ESR1 |
| GO-MF | GO:0016301 | kinase activity | 6 | 8.955224 | 0.001164 | PIK3CA, PLAU, AKT1, JAK2, EGFR, MTOR |
| GO-MF | GO:0043560 | insulin receptor substrate binding | 3 | 4.477612 | 0.001229 | PIK3CA, PTPN11, JAK2 |
| GO-MF | GO:0016757 | transferase activity, transferring glycosyl groups | 2 | 5.970149 | 0.001893 | GBA, ABO |
| GO-MF | GO:0019904 | protein domain specific binding | 6 | 8.955224 | 0.002264 | MDM2, RARA, PTPN11, PLG, RHOA, MTOR |
| GO-MF | GO:0005158 | insulin receptor binding | 3 | 4.477612 | 0.003165 | SRC, PTPN11, IGF1 |
| GO-MF | GO:0043274 | phospholipase binding | 3 | 4.477612 | 0.003432 | SRC, WAS, PTPN11 |
| GO-MF | GO:0051117 | ATPase binding | 4 | 5.970149 | 0.004238 | SRC, PDE4D, ESR1, EGFR |
| GO-MF | GO:0019903 | protein phosphatase binding | 4 | 5.970149 | 0.004238 | STAT3, PPARG, MET, EGFR |
| GO-MF | GO:0005516 | calmodulin binding | 5 | 7.462687 | 0.005767 | NOS2, NOS3, TRPV4, AKT1, EGFR |
| GO-MF | GO:0019838 | growth factor binding | 3 | 4.477612 | 0.007416 | KDR, TEK, TGFBR1 |
| GO-MF | GO:0004222 | metalloendopeptidase activity | 4 | 5.970149 | 0.008194 | MMP12, MMP2, MMP3, MMP9 |
| GO-MF | GO:0004190 | aspartic-type endopeptidase activity | 3 | 4.477612 | 0.00905 | BACE1, CASP3, REN |
| GO-MF | GO:0050661 | NADP binding | 3 | 4.477612 | 0.009481 | NOS2, NOS3, HMGCR |
| GO-MF | GO:0005006 | epidermal growth factor-activated receptor activity | 2 | 2.985075 | 0.010457 | ERBB4, EGFR |
| GO-MF | GO:0004517 | nitric-oxide synthase activity | 2 | 2.985075 | 0.010457 | NOS2, NOS3 |
| GO-MF | GO:0030331 | estrogen receptor binding | 3 | 4.477612 | 0.010828 | SRC, PPARG, ESR1 |
| GO-MF | GO:0008270 | zinc ion binding | 9 | 13.43284 | 0.010952 | MMP12, MMP2, ALB, MMP3, MDM2, RARA, PPARG, ESR1, MMP9 |
| GO-MF | GO:0004674 | protein serine/threonine kinase activity | 6 | 8.955224 | 0.011965 | MAPK10, PIK3CA, MAPK1, AKT1, TGFBR1, MTOR |
| GO-MF | GO:0090722 | receptor-receptor interaction | 2 | 2.985075 | 0.013919 | FGF2, FGFR1 |
| GO-MF | GO:0034617 | tetrahydrobiopterin binding | 2 | 2.985075 | 0.013919 | NOS2, NOS3 |
| GO-MF | GO:0044877 | macromolecular complex binding | 6 | 8.955224 | 0.014851 | TTR, CASP3, HTR2A, EPHB2, DRD2, MTOR |
| GO-MF | GO:0005080 | protein kinase C binding | 3 | 4.477612 | 0.016994 | SRC, TRPV4, AKT1 |
| GO-MF | GO:0005007 | fibroblast growth factor-activated receptor activity | 2 | 2.985075 | 0.017369 | FGFR2, FGFR1 |
| GO-MF | GO:0008236 | serine-type peptidase activity | 3 | 4.477612 | 0.018137 | F7, C1R, PLG |
| GO-MF | GO:0031625 | ubiquitin protein ligase binding | 5 | 7.462687 | 0.022275 | ABCB1, SRC, CBS, MDM2, EGFR |
| GO-MF | GO:0097110 | scaffold protein binding | 3 | 4.477612 | 0.02432 | SRC, NOS3, PDE4D |
| GO-MF | GO:0008237 | metallopeptidase activity | 3 | 4.477612 | 0.026321 | MMP2, MMP3, MMP9 |
| GO-MF | GO:1901363 | heterocyclic compound binding | 2 | 2.985075 | 0.03105 | PDE4D, DRD2 |
| GO-MF | GO:0034618 | arginine binding | 2 | 2.985075 | 0.03105 | NOS2, NOS3 |
| GO-MF | GO:0051428 | peptide hormone receptor binding | 2 | 2.985075 | 0.034441 | PTPN11, JAK2 |
| GO-MF | GO:0008134 | transcription factor binding | 4 | 5.970149 | 0.036832 | STAT3, RARA, PPARG, ESR1 |
| GO-MF | GO:0005003 | ephrin receptor activity | 2 | 2.985075 | 0.03782 | EPHB2, EPHB4 |
| GO-MF | GO:0030296 | protein tyrosine kinase activator activity | 2 | 2.985075 | 0.044543 | ERBB4, EGFR |
| GO-MF | GO:0019900 | kinase binding | 3 | 4.477612 | 0.049632 | SRC, PLG, EGFR |
| KEGG | hsa01521 | EGFR tyrosine kinase inhibitor resistance | 15 | 22.38806 | 3.71E-16 | SRC, STAT3, IGF1, FGF2, EGFR, MTOR, VEGFA, PIK3CA, KDR, AKT1, MAPK1, JAK2, MET, FGFR2, BCL2L1 |
| KEGG | hsa04015 | Rap1 signaling pathway | 18 | 26.86567 | 1.32E-13 | SRC, IGF1, FGF1, FGF2, RHOA, EGFR, VEGFA, PIK3CA, CNR1, KIT, KDR, AKT1, MAPK1, TEK, DRD2, MET, FGFR2, FGFR1 |
| KEGG | hsa04151 | PI3K-Akt signaling pathway | 21 | 31.34328 | 5.64E-13 | NOS3, IGF1, FGF1, FGF2, EGFR, IL2, MTOR, VEGFA, PIK3CA, ERBB4, KIT, MDM2, KDR, AKT1, MAPK1, TEK, JAK2, MET, FGFR2, FGFR1, BCL2L1 |
| KEGG | hsa04014 | Ras signaling pathway | 18 | 26.86567 | 9E-13 | PTPN11, IGF1, FGF1, FGF2, RHOA, EGFR, VEGFA, MAPK10, PIK3CA, KIT, KDR, AKT1, MAPK1, TEK, MET, FGFR2, FGFR1, BCL2L1 |
| KEGG | hsa01522 | Endocrine resistance | 12 | 17.91045 | 1.29E-10 | MAPK10, PIK3CA, SRC, MMP2, MDM2, MAPK1, AKT1, IGF1, ESR1, MMP9, EGFR, MTOR |
| KEGG | hsa04933 | AGE-RAGE signaling pathway in diabetic complications | 12 | 17.91045 | 1.61E-10 | MAPK10, PIK3CA, NOS3, CASP3, MMP2, STAT3, MAPK1, AKT1, JAK2, F3, TGFBR1, VEGFA |
| KEGG | hsa04010 | MAPK signaling pathway | 17 | 25.37313 | 3.26E-10 | IGF1, FGF1, FGF2, EGFR, TGFBR1, VEGFA, MAPK10, ERBB4, CASP3, KIT, KDR, AKT1, MAPK1, TEK, MET, FGFR2, FGFR1 |
| KEGG | hsa04926 | Relaxin signaling pathway | 12 | 17.91045 | 2.56E-09 | MAPK10, PIK3CA, NOS2, SRC, NOS3, MMP2, MAPK1, AKT1, MMP9, EGFR, TGFBR1, VEGFA |
| KEGG | hsa04066 | HIF-1 signaling pathway | 11 | 16.41791 | 7.14E-09 | PIK3CA, NOS2, NOS3, STAT3, MAPK1, AKT1, TEK, IGF1, EGFR, MTOR, VEGFA |
| KEGG | hsa04610 | Complement and coagulation cascades | 9 | 13.43284 | 2.12E-07 | F7, SERPINA1, F10, PLAU, C1R, F11, PLG, F2, F3 |
| KEGG | hsa04915 | Estrogen signaling pathway | 10 | 14.92537 | 8.23E-07 | PIK3CA, SRC, NOS3, MMP2, RARA, MAPK1, AKT1, ESR1, MMP9, EGFR |
| KEGG | hsa04917 | Prolactin signaling pathway | 8 | 11.9403 | 8.49E-07 | MAPK10, PIK3CA, SRC, STAT3, MAPK1, AKT1, JAK2, ESR1 |
| KEGG | hsa04520 | Adherens junction | 8 | 11.9403 | 9.36E-07 | SRC, WAS, MAPK1, MET, RHOA, EGFR, TGFBR1, FGFR1 |
| KEGG | hsa04625 | C-type lectin receptor signaling pathway | 9 | 13.43284 | 1.01E-06 | MAPK10, PIK3CA, SRC, MDM2, MAPK1, AKT1, PTPN11, RHOA, IL2 |
| KEGG | hsa04020 | Calcium signaling pathway | 12 | 17.91045 | 1.52E-06 | NOS2, ERBB4, NOS3, KDR, HTR2A, FGF1, FGF2, MET, EGFR, FGFR2, FGFR1, VEGFA |
| KEGG | hsa04510 | Focal adhesion | 11 | 16.41791 | 2.31E-06 | MAPK10, PIK3CA, SRC, KDR, MAPK1, AKT1, IGF1, MET, RHOA, EGFR, VEGFA |
| KEGG | hsa04012 | ErbB signaling pathway | 8 | 11.9403 | 3.2E-06 | MAPK10, PIK3CA, ERBB4, SRC, MAPK1, AKT1, EGFR, MTOR |
| KEGG | hsa04370 | VEGF signaling pathway | 7 | 10.44776 | 4.94E-06 | PIK3CA, SRC, NOS3, KDR, MAPK1, AKT1, VEGFA |
| KEGG | hsa04068 | FoxO signaling pathway | 9 | 13.43284 | 5.79E-06 | MAPK10, PIK3CA, STAT3, MDM2, MAPK1, AKT1, IGF1, EGFR, TGFBR1 |
| KEGG | hsa04550 | Signaling pathways regulating pluripotency of stem cells | 9 | 13.43284 | 1.11E-05 | PIK3CA, STAT3, MAPK1, AKT1, IGF1, JAK2, FGF2, FGFR2, FGFR1 |
| KEGG | hsa04072 | Phospholipase D signaling pathway | 9 | 13.43284 | 1.43E-05 | PIK3CA, KIT, MAPK1, AKT1, PTPN11, F2, RHOA, EGFR, MTOR |
| KEGG | hsa04659 | Th17 cell differentiation | 8 | 11.9403 | 1.58E-05 | MAPK10, STAT3, RARA, MAPK1, JAK2, TGFBR1, IL2, MTOR |
| KEGG | hsa04630 | JAK-STAT signaling pathway | 9 | 13.43284 | 2.75E-05 | PIK3CA, STAT3, AKT1, PTPN11, JAK2, EGFR, IL2, MTOR, BCL2L1 |
| KEGG | hsa04935 | Growth hormone synthesis, secretion and action | 8 | 11.9403 | 3.13E-05 | MAPK10, PIK3CA, STAT3, MAPK1, AKT1, IGF1, JAK2, MTOR |
| KEGG | hsa04810 | Regulation of actin cytoskeleton | 10 | 14.92537 | 3.47E-05 | PIK3CA, SRC, MAPK1, F2, FGF1, FGF2, RHOA, EGFR, FGFR2, FGFR1 |
| KEGG | hsa04931 | Insulin resistance | 7 | 10.44776 | 0.000157 | MAPK10, PIK3CA, NOS3, STAT3, AKT1, PTPN11, MTOR |
| KEGG | hsa04920 | Adipocytokine signaling pathway | 6 | 8.955224 | 0.000166 | MAPK10, STAT3, AKT1, PTPN11, JAK2, MTOR |
| KEGG | hsa04668 | TNF signaling pathway | 7 | 10.44776 | 0.000192 | MAPK10, PIK3CA, CASP3, MMP3, MAPK1, AKT1, MMP9 |
| KEGG | hsa01524 | Platinum drug resistance | 6 | 8.955224 | 0.000217 | PIK3CA, CASP3, MDM2, MAPK1, AKT1, BCL2L1 |
| KEGG | hsa04919 | Thyroid hormone signaling pathway | 7 | 10.44776 | 0.000292 | PIK3CA, SRC, MDM2, MAPK1, AKT1, ESR1, MTOR |
| KEGG | hsa04611 | Platelet activation | 7 | 10.44776 | 0.000334 | PIK3CA, SRC, NOS3, MAPK1, AKT1, F2, RHOA |
| KEGG | hsa04360 | Axon guidance | 8 | 11.9403 | 0.000426 | PIK3CA, SRC, MAPK1, PTPN11, EPHB2, MET, EPHB4, RHOA |
| KEGG | hsa04062 | Chemokine signaling pathway | 8 | 11.9403 | 0.000587 | PIK3CA, SRC, STAT3, WAS, MAPK1, AKT1, JAK2, RHOA |
| KEGG | hsa04750 | Inflammatory mediator regulation of TRP channels | 6 | 8.955224 | 0.000847 | MAPK10, PIK3CA, SRC, TRPV4, IGF1, HTR2A |
| KEGG | hsa04660 | T cell receptor signaling pathway | 6 | 8.955224 | 0.001108 | MAPK10, PIK3CA, MAPK1, AKT1, RHOA, IL2 |
| KEGG | hsa04218 | Cellular senescence | 7 | 10.44776 | 0.001124 | PIK3CA, TRPV4, MDM2, MAPK1, AKT1, TGFBR1, MTOR |
| KEGG | hsa04722 | Neurotrophin signaling pathway | 6 | 8.955224 | 0.002018 | MAPK10, PIK3CA, MAPK1, AKT1, PTPN11, RHOA |
| KEGG | hsa04071 | Sphingolipid signaling pathway | 6 | 8.955224 | 0.002018 | MAPK10, PIK3CA, NOS3, MAPK1, AKT1, RHOA 63 |
| KEGG | hsa04152 | AMPK signaling pathway | 6 | 8.955224 | 0.002171 | PIK3CA, AKT1, PPARG, HMGCR, IGF1, MTOR |
| KEGG | hsa04380 | Osteoclast differentiation | 6 | 8.955224 | 0.002775 | MAPK10, PIK3CA, MAPK1, AKT1, PPARG, TGFBR1 |
| KEGG | hsa04210 | Apoptosis | 6 | 8.955224 | 0.003604 | MAPK10, PIK3CA, CASP3, MAPK1, AKT1, BCL2L1 |
| KEGG | hsa04371 | Apelin signaling pathway | 6 | 8.955224 | 0.003957 | NOS2, NOS3, MAPK1, AKT1, TGFBR1, MTOR |
| KEGG | hsa04140 | Autophagy - animal | 6 | 8.955224 | 0.004206 | MAPK10, PIK3CA, MAPK1, AKT1, MTOR, BCL2L1 |
| KEGG | hsa04540 | Gap junction | 5 | 7.462687 | 0.004369 | SRC, MAPK1, HTR2A, DRD2, EGFR |
| KEGG | hsa04211 | Longevity regulating pathway | 5 | 7.462687 | 0.004549 | PIK3CA, AKT1, PPARG, IGF1, MTOR |
| KEGG | hsa04930 | Type II diabetes mellitus | 4 | 5.970149 | 0.005019 | MAPK10, PIK3CA, MAPK1, MTOR |
| KEGG | hsa04912 | GnRH signaling pathway | 5 | 7.462687 | 0.005319 | MAPK10, SRC, MMP2, MAPK1, EGFR |
| KEGG | hsa04657 | IL-17 signaling pathway | 5 | 7.462687 | 0.005524 | MAPK10, CASP3, MMP3, MAPK1, MMP9 |
| KEGG | hsa04024 | cAMP signaling pathway | 7 | 10.44776 | 0.00639 | MAPK10, PIK3CA, PDE4D, MAPK1, AKT1, DRD2, RHOA |
| KEGG | hsa04150 | mTOR signaling pathway | 6 | 8.955224 | 0.006439 | PIK3CA, MAPK1, AKT1, IGF1, RHOA, MTOR |
| KEGG | hsa04914 | Progesterone-mediated oocyte maturation | 5 | 7.462687 | 0.007359 | MAPK10, PIK3CA, MAPK1, AKT1, IGF1 |
| KEGG | hsa04928 | Parathyroid hormone synthesis, secretion and action | 5 | 7.462687 | 0.008412 | PDE4D, MAPK1, RHOA, EGFR, FGFR1 |
| KEGG | hsa04670 | Leukocyte transendothelial migration | 5 | 7.462687 | 0.010804 | PIK3CA, MMP2, PTPN11, MMP9, RHOA |
| KEGG | hsa04213 | Longevity regulating pathway - multiple species | 4 | 5.970149 | 0.011478 | PIK3CA, AKT1, IGF1, MTOR |
| KEGG | hsa04613 | Neutrophil extracellular trap formation | 6 | 8.955224 | 0.014357 | PIK3CA, SRC, MAPK1, AKT1, ELANE, MTOR |
| KEGG | hsa04664 | Fc epsilon RI signaling pathway | 4 | 5.970149 | 0.01473 | MAPK10, PIK3CA, MAPK1, AKT1 |
| KEGG | hsa04115 | p53 signaling pathway | 4 | 5.970149 | 0.017799 | CASP3, MDM2, IGF1, BCL2L1 |
| KEGG | hsa04910 | Insulin signaling pathway | 5 | 7.462687 | 0.019994 | MAPK10, PIK3CA, MAPK1, AKT1, MTOR |
| KEGG | hsa04215 | Apoptosis - multiple species | 3 | 4.477612 | 0.024319 | MAPK10, CASP3, BCL2L1 |
| KEGG | hsa04921 | Oxytocin signaling pathway | 5 | 7.462687 | 0.029182 | SRC, NOS3, MAPK1, RHOA, EGFR |
| KEGG | hsa04932 | Non-alcoholic fatty liver disease | 5 | 7.462687 | 0.029789 | MAPK10, PIK3CA, CASP3, AKT1, PPARG |
| KEGG | hsa04960 | Aldosterone-regulated sodium reabsorption | 3 | 4.477612 | 0.031875 | PIK3CA, MAPK1, IGF1 |
| KEGG | hsa04658 | Th1 and Th2 cell differentiation | 4 | 5.970149 | 0.032488 | MAPK10, MAPK1, JAK2, IL2 |
| KEGG | hsa04666 | Fc gamma R-mediated phagocytosis | 4 | 5.970149 | 0.037147 | PIK3CA, WAS, MAPK1, AKT1 |
| KEGG | hsa04144 | Endocytosis | 6 | 8.955224 | 0.041281 | SRC, MDM2, RHOA, EGFR, TGFBR1, FGFR2 |
| KEGG | hsa04620 | Toll-like receptor signaling pathway | 4 | 5.970149 | 0.044216 | MAPK10, PIK3CA, MAPK1, AKT1 |
